# On the engineering of reductase-based-monooxygenase activity in CYP450 peroxygenases†

**DOI:** 10.1039/d3sc06538c

**Published:** 2024-03-07

**Authors:** Shalini Yadav, Sason Shaik, Kshatresh Dutta Dubey

**Affiliations:** a Department of Chemistry, School of Natural Science, Shiv Nadar Institution of Eminence NH91 Tehsil Dadri Greater Noida Uttar Pradesh 201314 India kshatresh.dubey@snu.edu.in; b Institute of Chemistry, The Hebrew University Edmond J. Safra Campus at Givat Ram Jerusalem 9190401 Israel sason@yfaat.ch.huji.ac.il

## Abstract

Recent bioengineering of CYP450_OleT_ shows that peroxide-based CYP450_OleT_ can be converted to a reductase-based self-sufficient enzyme, which is capable of showing efficient hydroxylation and decarboxylation activity for a wide range of substrates. The so-generated enzyme creates several mechanistic puzzles: (A) as CYP450 peroxygenases lack the conventional acid–alcohol pair, what is the source of two protons that are required to create the ultimate oxidant Cpd I? (B) Why is it only CYP450_OleT_ that shows the reductase-based activity but no other CYP members? The present study provides a mechanistic solution to these puzzles using comprehensive MD simulations and hybrid QM/MM calculations. We show that the fusion of the reductase domain to the heme-binding domain triggers significant conformational rearrangement, which is gated by the propionate side chain, which constitutes a new water aqueduct *via* the carboxylate end of the substrate that ultimately participates in Cpd I formation. Importantly, such well-synchronized choreographies are controlled by remotely located Tyr359, which senses the fusion of reductase and communicates to the heme domain *via* non-covalent interactions. These findings provide crucial insights and a broader perspective which enables us to make a verifiable prediction: thus, the catalytic activity is not only limited to the first or second catalytic shell of an enzyme. Furthermore, it is predicted that reinstatement of tyrosine at a similar position in other members of CYP450 peroxygenases can convert these enzymes to reductase-based monooxygenases.

## Abstract 2

Recent bioengineering of CYP450_OleT_ shows that peroxide-based CYP450_OleT_ can be converted to a reductase-based self-sufficient enzyme, which is capable of showing efficient hydroxylation and decarboxylation activity for a wide range of substrates.

## Graphical abstract

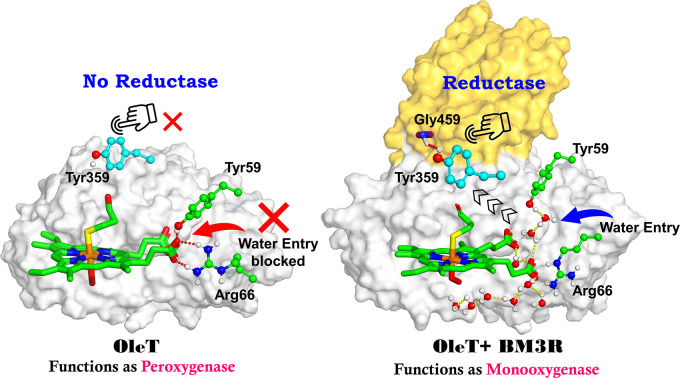

## 1. Introduction

The production of hydrocarbons in nature is restricted to a limited number of organisms.^1–7^ CYP450_OleT_ (CYP152L1 from *Jeotgalicoccus* sp. ATCC 8456), a member of CYP450 (Cytochrome 450) peroxygenase superfamily, is among such rare enzymes that have evolved to produce aliphatic hydrocarbons (α-alkenes) by the decarboxylation of the fatty acid substrate.^8,9^ Aliphatic α-alkenes are important components that can be used to replace fossil fuels, as well as for the production of lubricants, detergents, and polymers.^10–12^ This makes CYP450_OleT_ a promising biocatalyst for biofuel production and commercial applications.

**Scheme 1:**
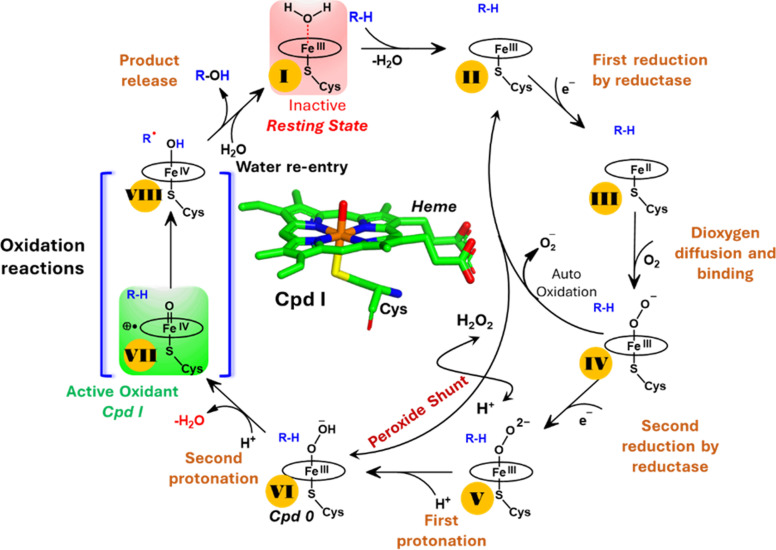
A generic catalytic cycle for CYP450 enzymes using O_2_, 2e^−^, and H^+^ (steps: I → II → III → IV → V → VI → VII) and using directly H_2_O_2_ (peroxide shunt) as a co-substrate to generate the active oxidant Cpd I by the CYP450 peroxygenase family.

Unlike typical CYP450 monooxygenases that use molecular O_2_ and redox partners, CYP450_OleT_ uses H_2_O_2_ as the native oxygenating source as can be seen in Scheme 1.^13^ Although H_2_O_2_ is much cheaper than NADPH and other redox proteins, the large-scale production of α-alkenes demands high concentrations of H_2_O_2_ which constitute environmental hazards and cause deactivation of the oxidant of the biocatalyst. For this reason, Liu *et al.* used an alternative redox partner, RhFRED from *Rhodococcus.* sp., and observed that, even without H_2_O_2_ and presumably using O_2_, peroxygenases still catalyze efficiently the decarboxylation of fatty acids, with carbon chain lengths of C_12_ to C_20_.^14^ In a similar study, Lu *et al.* showed that the fusion of CYP450_OleT_ with the reductase domain of bacterial CYP450_BM3_ creates a self-sufficient enzyme, OleT-BM3R, capable of producing terminal alkenes from fatty acids of varying lengths using molecular O_2_.^15^ They further found that the fused OleT-BM3R enzyme has enhanced thermal stability compared to the isolated reductase domain BM3-R without CYP450_OleT_. As such, these results demonstrate that CYP450_OleT_ can function as a biocatalyst, similar to a conventional cytochrome P450, reducing O_2_ instead of using hydrogen peroxide to generate the reactive intermediate in its catalytic cycle.

Nevertheless, this creates several questions. CYP450 monooxygenases require two electrons and two protons to form the ultimate oxidant Cpd I (*cf.*Schemes 1 and S1†).^16–19^ The two electrons are normally supplied by the reductase partner,^20–22,55^ while the two protons are provided by an organized water chain that is directed to the active site *via* acid–alcohol pairs (*cf.* states V and VI in Scheme 1).^23–27^ For example, in CYP450_BM3_, the acid–alcohol pair is Glu267–Thr268.^16,25^ However, the experimental 3D structures of CYP450 peroxygenases do not reveal any acid–alcohol pairs in the respective heme domains.^13^ This creates therefore the following mechanistic puzzle: (a) if CYP450 peroxygenases lack the conventional acid–alcohol pair, then what could be the possible protonation routes for the CYP450 peroxygenase in the fused CYP450_OleT-BM3R_ complex to form the ultimate oxidant Cpd I? (*cf.* Scheme S2†). (b) Why is CYP450_OleT_ the only peroxygenase family member, which exhibits such reductase-based monooxygenase activity? (c) Can we reproduce similar reductase-based monooxygenase activity in other members as well?

The present study addresses these issues using comprehensive MD simulation and hybrid QM/MM calculations. We show here that the fusion of the reductase domain to the heme binding domain triggers a well-synchronized large conformational rearrangement *via* the propionate side chain which forms a new water route through the carboxylate end of the substrate. Thus, the substrate instigates the formation of Cpd I that eventually causes its own oxidation. Such well-synchronized choreographies are mastered by far-located Tyr359, which can sense the fusion of reductase and communicates this to the heme domain *via* non-covalent interactions. Interestingly, unlike other CYP450 peroxygenase members, CYP450_OleT_ possesses the key tyrosine 359 residue. As such, we propose that a similar reinstatement of the key residue may endow other members, as well, with a reductase-based monooxygenase activity.

## 2. Computational method and details

### 2.1 System preparation

**Fig. 1:**
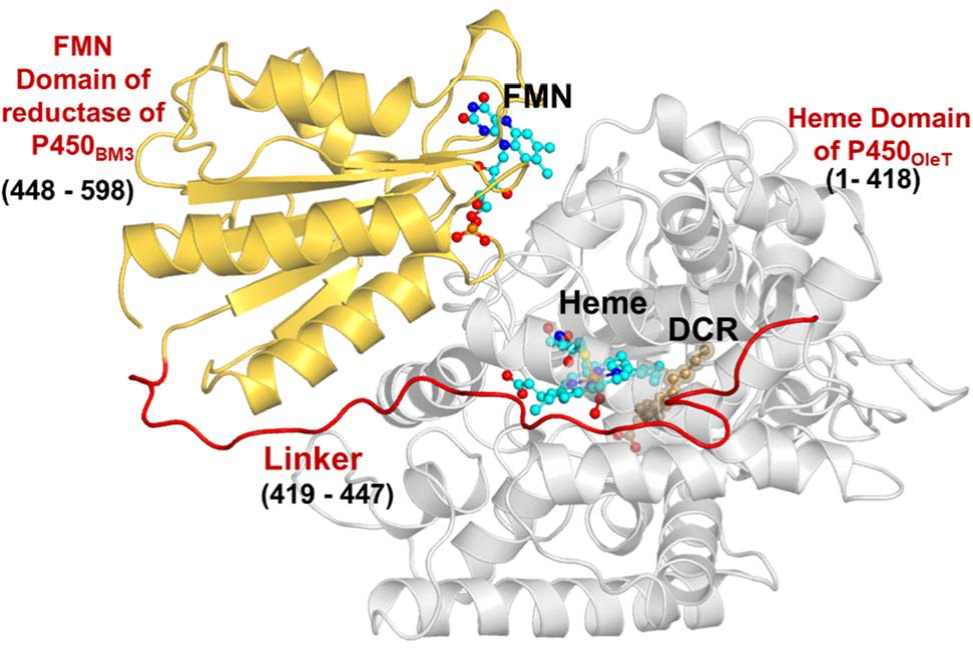
The fused protein, OleT-BM3R, includes the heme domain (in grey) and the FMN binding domain (in gold), which are linked *via* the red linker. The DCR (the three letter code for the substrate icosanoic acid) substrate, heme, and FMN are drawn as ball and stick models in brown and light blue, respectively.

The initial structure of the substrate bound heme-binding domain of CYP450_OleT_ (PDB entry: 4L40)^9^ and the FMN reductase domain of CYP450_BM3_ (PDB entry: 1BVY)^28^ were taken from the protein data bank. The crystal structures of CYP450_OleT_ and CYP450_BM3_ were superimposed using Chimera software,^29^ and the BM3 heme domain was deleted. The remaining combined CYP450_OleT_-FMN structure was subsequently used as a single PDB (*cf.*Fig. 1).

The two domains were further combined *via* a linker (provided in 1BVY) using MODELLER.^30^ Missing hydrogen atoms were added by the leap module of the Amber18 package. As our primary goal is to identify protonation pathways, we have considered only protonated residues that are in close proximity to the heme.

Force field parameters for a dioxygen heme complex were taken from already published data by Cheatham and co-workers.^31^ For the ferric-hydroperoxide species (Cpd 0), we generated parameters using the Metal Center Parameter Building (MCPB)^32^ module in Amber 18. The partial atomic charges and missing parameters for the substrate (DCR) and FMN were obtained from the RESP^33,34^ charge method at the HF/6-31G* level. Na^+^ ions were added to neutralize the system. Finally, the resulting system was solvated within an octahedral box of TIP3P^35^ water extending up to a minimum cutoff of 12 Å from the protein boundary. The Amber ff14SB^36^ force field was used for protein in all the simulations performed.

### 2.2 MD simulation

After complete parametrization, the geometry of the entire system was minimized (5000 steps of steepest descent and 5000 steps of conjugate gradient) to find the local energy minimum of the structure and to remove poor contacts. The post minimization system was gently annealed under the NVT ensemble for 50 ps with a weak restraint of 4 kcal mol^−1^ Å^−2^.

Subsequently, the system underwent 1 ns of density equilibration under the NPT ensemble at constant temperature (300 K) and pressure (1 atm), using the Langevin thermostat^37^ with a collision frequency of 2 ps, and the Berendsen barostat^38^ with a pressure relaxation time of 1 ps. This 1 ns density equilibration is a weakly restrained MD simulation, in which the system is released slowly to achieve a uniform density after heating under periodic boundary conditions.

After 1 ns of density equilibration, we removed all the afore-applied restraints (during heating and density equilibration) and the system underwent further equilibration for 3 ns to get a well-settled pressure and temperature for conformational and chemical analyses. This was followed by a MD production run for each system.

During all the MD simulations, covalent bonds containing hydrogens were constrained using the SHAKE^39^ algorithm, and the particle-mesh Ewald (PME)^40^ method was used to treat long-range electrostatic interactions. All the MD simulations were performed with the GPU version of the Amber 18 package.^41^

To understand this yet unclear source of protonation and conversion of dioxygen-state → Cpd 0 → Cpd I, MD simulation of 500 ns was performed on the following four systems, by using random velocity: (A) the peroxo-ferric state of the heme domain associated with the reductase domain; (B) the peroxo-ferric state of the heme domain only; (C) the Cpd 0 (ferric hydroperoxide) state of the heme domain associated with the reductase domain; (D) the Cpd 0 (ferric hydroperoxide) state of the heme domain only. Since our main objective is to find out a protonation source, we have kept all the titratable amino acid residues close (up to 5 Å) to the heme moiety in their protonated form, during all the simulation processes.

### 2.3 MMGBSA calculations

MMGBSA is a widely used method for studying protein–ligand interactions and protein–protein interactions. Here, the calculation was performed using the MMPBSA.py^51^ module of the AMBER simulation package.^41^ To check the energetics of the dynamic event that occurs after 200 ns, we have performed simulation in two sets. One set includes the geometry of protein before 200 ns and the second set involves the protein geometry after 200 ns. Water and ions were removed, and the dielectric constant was set to 1 for the solute and 80 for the solvent. The method combines molecular mechanics energy calculations, the solvent-accessible surface area, and the GB model (MMGBSA) to calculate solvation free energy.

### 2.4 QM/MM methodology

The mechanism for generating Cpd I from the peroxo-ferric species, Fe–OO^2−^ → Cpd 0 → Cpd I, was investigated by QM/MM calculations on representative snapshots from the MD trajectory. The calculations were carried out using ChemShell^42,43^ employing Turbomole^44^ for the QM part and DL_POLY^45,46^ using the FF14SB amber force field for the MM part.

The employed QM region contained the heme, the proton donor residues, and the water molecules, which shuttle protons towards the peroxo species. To account for the effect of the environment, during the QM/MM calculations, we included all the protein residues and water molecules present up to 8 Å of the heme surface, in the active region. The atoms of this active region interact with the QM atoms and lead to subsequent polarization effects through electrostatic and van der Waals interactions. An electronic embedding scheme was used to account for the polarizing effect of the protein residues in the QM region. Hydrogen link atoms with the charge-shift model^36^ were applied to treat the QM-MM boundary.

The QM region was computed by use of the UB3LYP^47^ functional with two basis sets Def2-SVP (denoted as B1) and Def2-TZVP (denoted as B2). B1 was used for geometry optimization, potential energy surface scanning, and frequency calculations. The transition states (TSs) were located by relaxed potential energy surface scans followed by full TS optimization using the partitioned rational function optimization (P-RFO)^48^ method implemented in the HDLC code. The zero-point energy was calculated at the B1 level, for all the species. Grimme dispersion (G-D3)^49^ was used to incorporate the dispersion correction in energetics. Energies were subsequently corrected by use of B2, and final energies are reported as UB3LYP/B2-D3+ZPE.

## 3. Results and discussion

### 3.1 Conformational dynamics in the heme binding domain (HBD) due to fusion of reductase

The study was initiated with simulation of the CYP450_OleT-BM3R_ complex in the heme-peroxo-ferric state. The linker loop (Fig. 1) was found to be the most flexible region during the simulations (Root Mean Square Fluctuation ∼4–6 Å in Fig. S1 and S2†). Additionally, during the simulation, the distance between the FMN and heme domains shrunk.

**Fig. 2:**
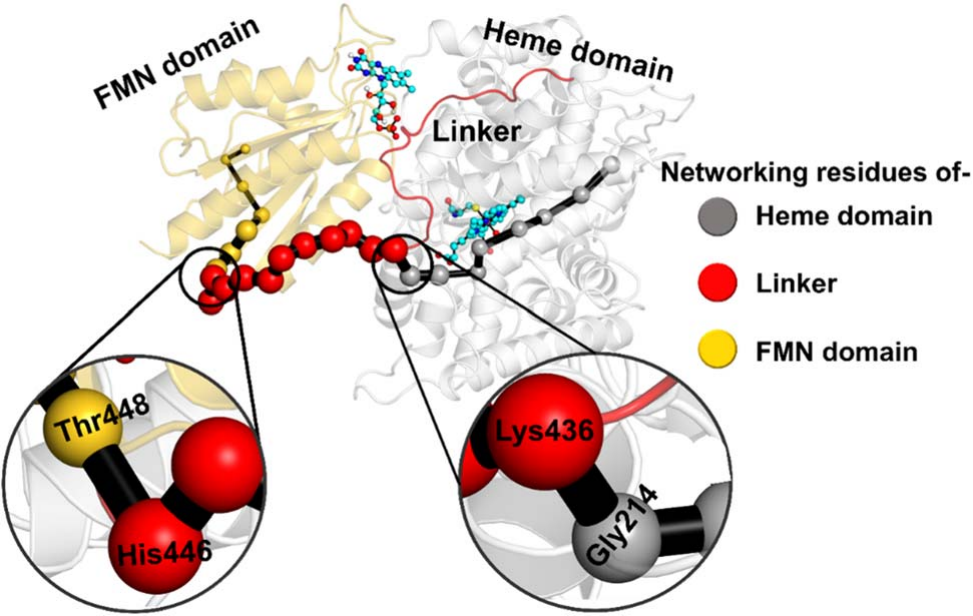
Representation of the shortest path map (SPM) between the heme domain (grey) of OleT and the FMN domain (gold) of reductase. Key portions are magnified. Beads in grey show the portion belonging to the heme domain, gold the portion of the FMN domain and red the portion of the linker loop. Note that the size of the beads indicates the strength of the interaction, while the black edges represent the communication path among the residues.

**Fig. 3:**
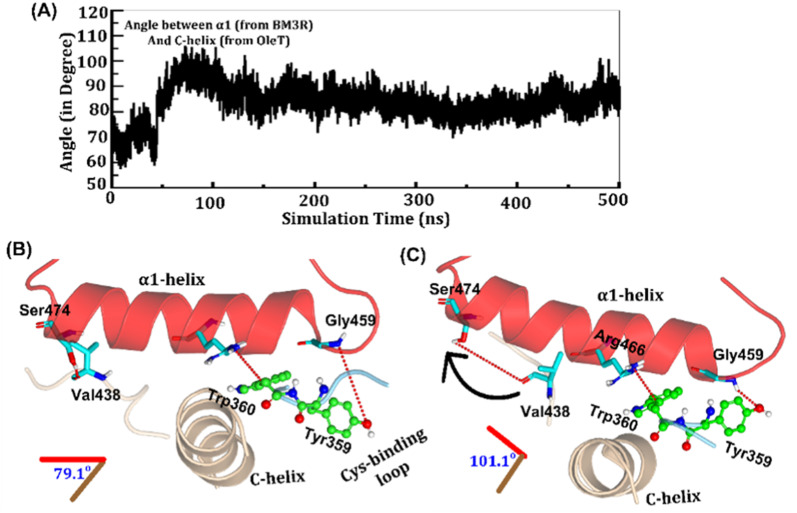
(A) Variation of the angle between α1-helix and C-helix during the MD simulations. (B and C) The key interdomain interactions where B shows initial conformation, while C is the final positioning of the C-helix, cysteine binding loop and α1-helix.

What factors drive the two domains to come closer to each other? To answer this question, we calculated the shortest path map (SPM),^50^ a correlation-based approach to study the allosteric communications between two distant substructures. The shortest communication path is shown in Fig. 2 as beads (as small spheres) and threads (as black), where the size of the beads shows the magnitude of the interaction while the threads (in black) show the residues participating in the communications. As can be seen, the linker loop (shown in red), is key to the inter-domain talk that connects Gly214 of the heme domain to His446 of the FMN/reductase domain. We believe that this interaction network is crucial for bringing the two domains closer to each other during the simulations. In addition, the angle between the centers of mass of the HBD and the reductase also changes with the simulations (Fig. 3A), indicating topological rearrangement in the HBD due to reductase attachment. Our simulations show that this conformational change is primarily orchestrated by the C-helix of the HBD and the α_1_ helix of BM3-R.

As shown in Fig. 3A–C, the helix angle between C and α1 helices changes from ∼79° to 101° and remains constant, throughout the simulation. We found that the interdomain interactions of Val438_HBD_ with Ser474_BM3-R_ and Tyr359_HBD_ with Gly459_BM3-R_ (subscript HBD is for the heme domain and BM3-R is for the reductase domain) are key for this change in the helix angle. As can be seen, initially (Fig. 3B), Gly459 of the α1-helix is far away (∼7.1 Å) from the Tyr359 residue of the heme domain. However, at the later stage of simulation (Fig. 3C), the same Gly459 moves closer to Tyr359 and forms a strong hydrogen bond with Tyr359. We believe that this alteration in the interaction between Gly450 and Tyr359 is the root cause of the tilting of C and α1 helices. The energetic calculations of the two different conformations *i.e.*, Fig. 3B and C, also show that the final conformations of C and α1 (*i.e.*, Fig. 3C) are stabilized by ∼33 kcal mol^−1^ relative to the initial conformation (see Table S2† for the MMPBSA analysis).^51^ The thermodynamic stability of conformation 3B by ∼33 kcal mol^−1^ provides the driving force needed for the change of orientation of the C and α1-helices. In the subsequent section, we will see that this conformational change due to the Tyr359–Gly459 interaction works as a signal that triggers the necessary water inflow into the heme site and propagates the catalytic cycle.

### 3.2 The mechanism of water inflow into the heme active site

Water inflow is crucial in conventional CYP450 monooxygenases for proton shuttling during the catalytic cycle,^52^ and it has been documented in several articles; however, the formation of a proton channel in engineered CYP450_OleT_ fused with reductase is nevertheless intriguing to study. Therefore, we performed two separate sets of simulations: (a) without fused reductase and (b) with fused reductase, and we carefully monitored the enzyme conformation *via* MD simulations to study the pattern of water inflow. In (a) without reductase, we did not see, during the entire 500 ns of simulation, much water inflow *via* the propionate side chain as is necessary for the first protonation (heme-peroxo → Cpd 0). However, when the reductase was included in (b), there appeared a consistent water inflow after ∼200 ns of the simulation (see Fig. S3†).

**Fig. 4:**
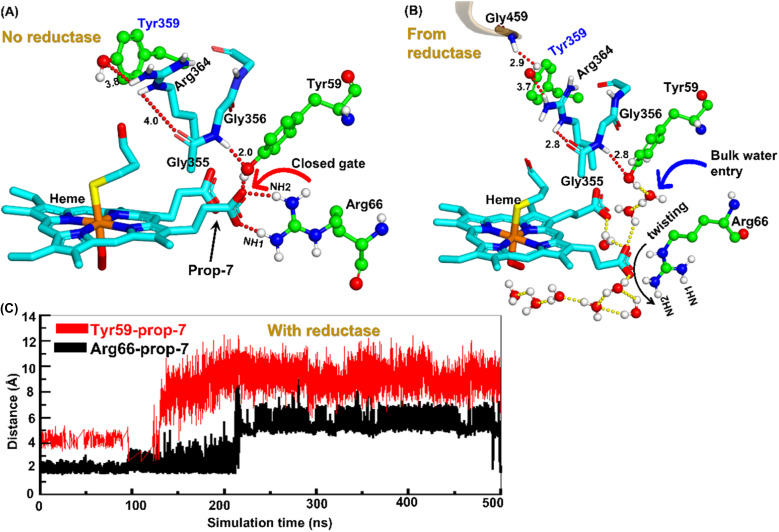
Formation of an ordered H-bond network between residues Tyr359 of the heme domain to heme prop7 (A) before reductase attaches and (B) after reductase attaches. Note here how a new interaction between Gly459 of the reductase with Tyr359 perturbs the entire H-bond network after the fusion of reductase. (C) A plot showing the increasing Prop7–Tyr59 and Prop7–Arg66 distances, which signifies a gate opening.

To unravel the mechanism of water inflow, we carefully looked at the active site topology of the heme-binding domain in both cases, and we found that the conformation of the propionate side chain (prop7), Tyr59 and Arg66 is crucial for the water flow. In the absence of reductase, both the guanidium hydrogen atoms of Arg66 and the hydroxy group of Tyr59 interact strongly with prop7 of the heme prosthetic group (see Fig. 4A). These interactions form a closed doorway that blocks the water egress through heme-peroxo. Interestingly, in case (b), where reductase is fused, Tyr59 and Arg66 move apart and open a water channel that stretches through the heme-peroxo *via* propionate (Fig. 4B).

So how does the enzyme sense the opening of the water gateway when the reductase binds to the heme-binding domain? We found that residue Tyr359 is the key and the transducer to open and close the water gate by releasing a signal through non-covalent interactions. When there is no reductase, Tyr359 is weakly associated with the interaction network of Tyr359–Arg364–Gly354–Gly355–Tyr59–Prop7. However, when the reductase gets attached, it undergoes a conformational rearrangement, which is thermodynamically driven by the Gly459–Tyr359 interaction (see the previous section and a discussion on the movement of the C-helix due to reductase attachment), thus pulling the interaction network towards Tyr359, and thereby opening the gate for water inflow.

In summary, the interaction between the two domains in the fused protein OleT-BM3R disrupts the H-bond interaction between the heme propionate and the Tyr59 and Arg66 residues in the ferric-peroxo species of heme. This disruption leads to the opening of the gate *via* Prop7, which in turn enables bulk water to enter the active site *via* the distal site of the heme domain *i.e.*, from the FMN domain (see also Fig. S4† for the loop dynamics).

### 3.3 Why is P450_OleT_ unique in showing the reductase-based monooxygenase activity?

**Fig. 5:**
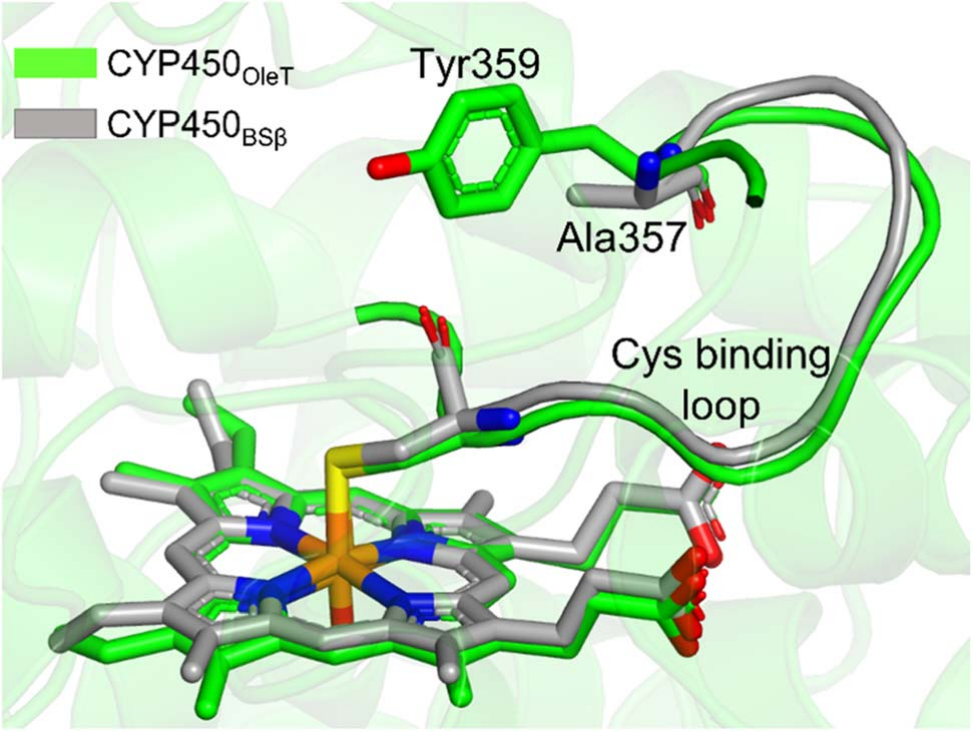
The superimposed structure of CYP450_OleT_ with CYP450_BSβ_. Note the lack of Tyr359 in CYP450_BSβ._

It is important to note, CYP450_OleT_ is unique in the peroxygenase family that shows the reductase-based monooxygenase activity. The similar experiments for the fusion of the reductase with other members of the peroxygenase family, such as in CYP450_BSβ_ (CYP152A1 from *Bacillus subtilis*), did not appear to function for their native reactions (β-hydroxylation of fatty acid).^53^ To investigate the root cause we inspected the availability of the key residues in all three members. Interestingly, as shown in Fig. 5, CYP450_BSβ_ lacks Tyr359 or an analogous residue, which can sustain water inflow. As mentioned above, Tyr359 is the key residue that opens the gate to facilitate the water inflow in CYP450_OleT_. Thus, the absence of Tyr359 in CYP450_BSβ_ prevents the water inflow and consequently, CYP450_BSβ_ does not show reductase-based monooxygenase activity. This provides solid experimental support of the role of Tyr359 in the reductase activity.

### 3.4 The protonation route in CYP450_OleT_ fused with an FMN domain

The water inflow is crucial for the first protonation of the heme-peroxo complex. In previous studies it is found that the first protonation could be facilitated *via* propionate or other sources than an acid–alcohol pair.^25^ Therefore, we carefully monitored the different water pathway that is connected with a possible base.

**Fig. 6:**
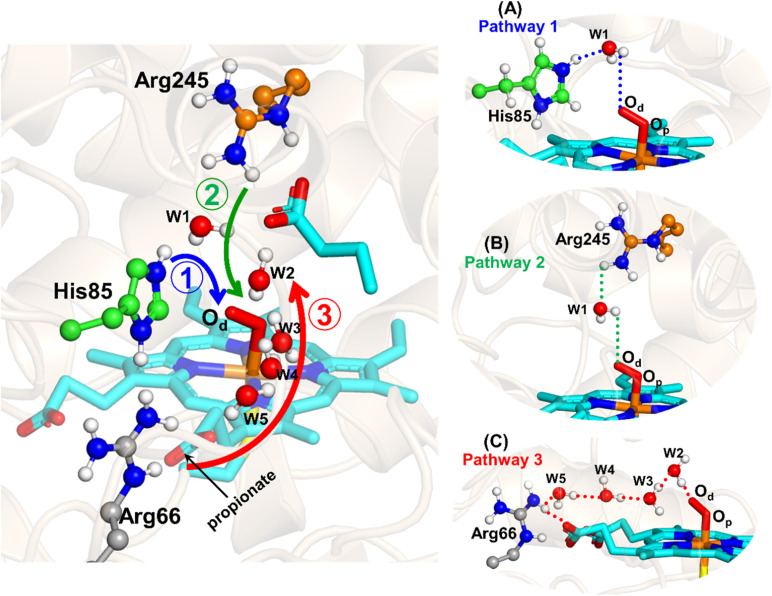
Hydrogen bonding network between water and potential proton–donor amino acid residues, which connect the distal oxygen (O_d_) of heme. Note that the representative snapshot has been taken from the most populated frame obtained from cluster analysis of a 500 ns MD trajectory. (A–C) The three possible pathways (shown by the three different colored residues) for plausible proton transfer to the distal oxygen (O_d_) to form Cpd 0 through Arg66/His85/Arg245.

As can be seen from Fig. 6, there are three possible water networks present in the active site, which can be potential proton donors to the distal oxygen (O_d_) in order to convert dioxygen Fe(iii) to Cpd 0, *i.e.*, state 5 → 6: (i) protonation through His85 → (H_2_O) → O_d_; (ii) protonation through Arg245 → (H_2_O) → O_d_; (iii) protonation through Arg66 → (H_2_O)_*n*_ → O_d_. To check the respective potencies of protonation through these residues, we have performed QM/MM calculation.

#### 3.4.1 Mechanism of the first protonation *via* QM/MM calculations

Unlike classical MD simulations, which cannot handle bond breaking/making, hybrid QM/MM calculations can explore the mechanism of Cpd 0 formation in fused OleT-BM3R. Here, initially, we investigated the mechanism of the first protonation *i.e.*, the formation of the oxidant Cpd 0. In doing so we have explored all three possible pathways to generate Fe–OO^2−^ → Fe–OOH^−^. As can be seen in Fig. 6, the distal oxygen (O_d_) of the dioxygen moiety is connected to His85 and Arg245 *via* a common water molecule w1 (*cf.*Fig. 6A and B). Meanwhile in the other plausible pathway, Arg66 connects the distal oxygen (O_d_), *via* several water molecules (*cf.*Fig. 6C). For a clear observation we have performed QM/MM separately for all three possibilities.

**Fig. 7:**
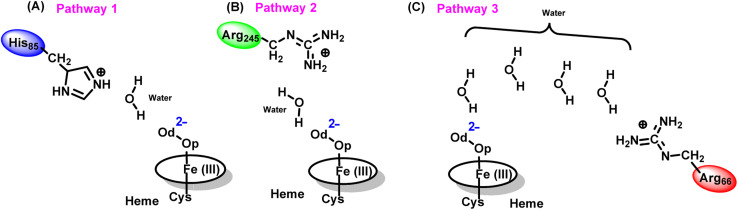
QM region selected for all three pathways (A to C) to proceed the QM/MM calculation.

**Fig. 8:**
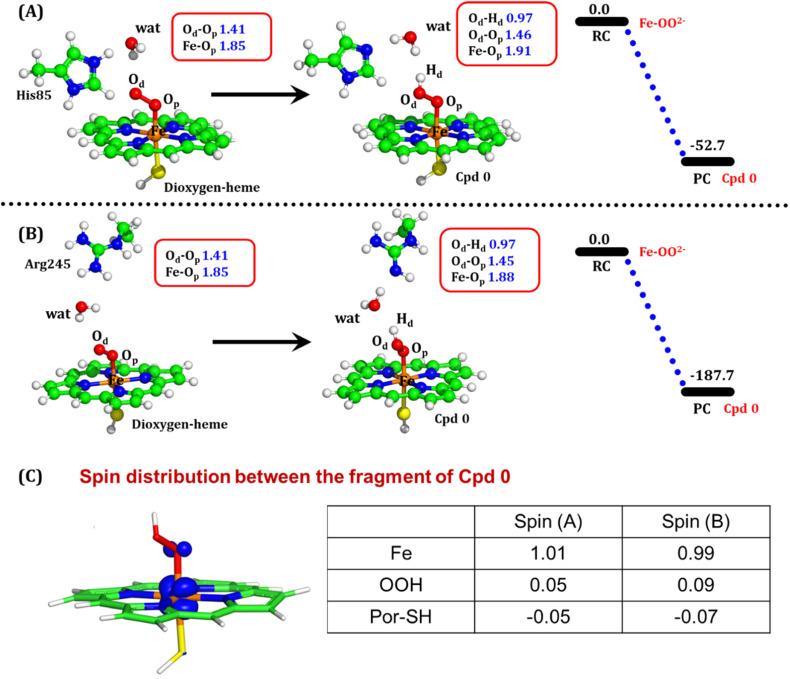
QM/MM optimized geometries for the Cpd 0 formation when (A) His85 acts as a proton donor and (B) Arg245 acts as a proton donor. (C) Electronic spin distribution of the Cpd 0 state. Blue isosurface depicts the presence of one alpha electron on Fe. Note that the energies are reported at the B2-D3 + ZPE level of theory. All energies are in kcal mol^−1^.

To get the respective reaction coordinates and energy profiles for Pathways 1 and 2, we performed the potential energy surface (PES) scanning of the QM/MM optimized geometries. The QM regions used for each pathway are shown in Fig. 7A–C. Interestingly, in the paths A and B, the dioxygen moiety of ferric-peroxide species (strongly basic in nature) accepts the proton from the nearby source His85 and Arg245 in a barrierless manner and generates Cpd 0 (see Fig. 8A and B). Meanwhile the third pathway, which is supposed to be facilitated *via* distant Arg66 through organized water chains, is found to be highly unfavourable for Cpd 0 formation (*e.g.*, the energy barrier is more than 32.5 kcal mol^−1^, see Fig. S5† for details). This indicates that Arg66 may not directly participate in the proton transfer mechanism, but instead, it serves as a gatekeeper to facilitate the entry of water molecules towards the active site, as we discussed in an earlier section (*cf.*Fig. 6). In essence, while Arg66 is not directly involved in the conversion of dioxygen to Cpd 0, its role is pivotal in facilitating water entry to the heme active site to promote the process.

To comprehend the electronic structure of the resulting product (Cpd 0), the spin populations of crucial atoms and groups were computed. In both pathways, the total spin densities are comparable, with the Fe center having one unpaired electron with an occupancy of approximately 1.0. This is a typical characteristic of Cpd 0 states observed in CYP450 enzymes.^16,18^

### 3.5 MD result of OleT-BM3R in the ferric hydroperoxide Fe–OOH– state

**Fig. 9:**
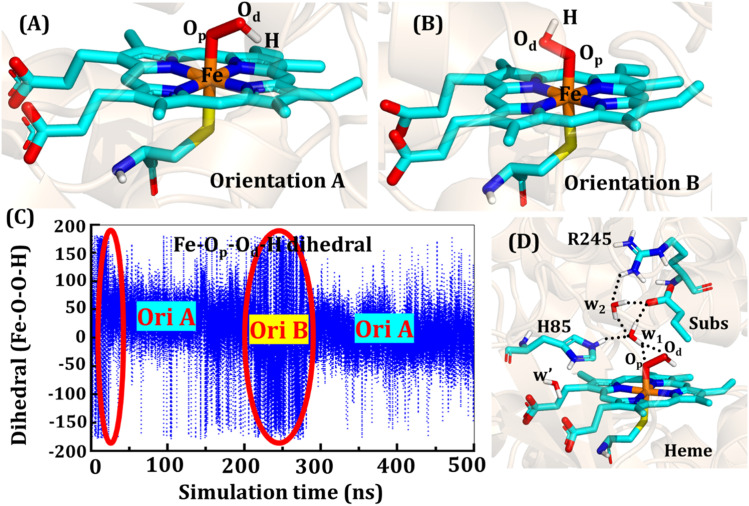
Two prominent orientations (A and B) of the hydroperoxide moiety in Cpd 0. (C) Dihedral angle of Fe–O_p_–O_d_–H showing the rotation of OOH in the active site. The area encircled by the red ellipsoids shows the less prominent conformation in the time span of 500 ns. (D) Active site network near the active site of heme in the Cpd 0 state.

The second protonation occurs at Cpd 0, which is commonly thought to be a necessary step for the eventual formation of the active oxidant Cpd I. Furthermore, though the Cpd I formation from Cpd 0 in cysteine-ligated conventional CYP450s is well established,^16,24–26^ the mechanism of Cpd I formation through Cpd 0 in peroxygenase CP450 is yet to be elucidated. In doing so, we performed 500 ns of MD simulations (along with 2 replicas; 2 × 500 ns) of OleT-BM3R in the Cpd 0 state of heme. The root mean square deviation of the system converged after a simulation time of about 70 ns (*cf.* Fig. S6†). In all the simulations, the hydroperoxide (OOH–) moiety of heme was flexible and rotated freely in two possible orientations A and B as shown in Fig. 9A and B. This flexibility and rotation ability of the OOH– moiety can be anticipated from the drastic changes (+180° to −180°) in the dihedral angle of Fe–O_p_–O_d_–H in Cpd 0 of OleT-BM3R (*cf.*Fig. 9C).

Similar to the ferric-peroxide state, a careful inspection of the trajectory of the active site (near to Fe–OOH^−^) shows a consistent water flow, *via* propionate. This flow is maintained throughout the 500 ns simulation time in the Cpd 0 state (*cf.* Fig. S7†). Surprisingly, in addition to ferric-peroxide, Cpd 0 also exhibits a disruption of the salt bridge between Arg66 and the propionate group, as shown in Fig. S8,† which reinforces the idea of Arg66 being a regulator of the water gate. Upon examining the hydrogen bonding network near the Fe–OOH– site, we noted the presence of the amino acid residues His85 and Arg245, as well as the substrate interacting with the hydroperoxide moiety, as depicted in Fig. 9D.

As can be seen in Fig. 9D, the presence of two water molecules close to the OOH– moiety connects His85, Arg245, and the substrate (DCR). Thus, water w_1_ is connected with the imidazolium side chain of His85 and both oxygens (proximal O_p_ and distal O_d_) of the hydroperoxide of the heme moiety. On the other side, Arg245 and substrate's carboxylic acid end are connected to the heme *via* water w_1_ and w_2_. This interaction persists for most of the simulation time. Since protonation *via* water molecules is more common in P450 chemistry,^16^ we have considered the amino acid residue of the H-bonding network for the protonation source. In this state, we have tested the nearest protonation source: (a) protonation *via* His85; (b) protonation *via* substrate carboxylic acid; (c) protonation *via* Arg245.

#### 3.5.1 The mechanism of Cpd I formation

The second protonation at Cpd 0 leads to dehydration (removal of one water molecule) and Cpd I formation. In the previous section, as we have seen that, close to the active site (Fe–OOH^−^) the hydrogen bonding network still suggests that for His85, Arg245 or the substrate might act as a proton donor source for Cpd 0.

#### 3.5.2 Pathway 1: protonation *via* His85

**Fig. 10:**
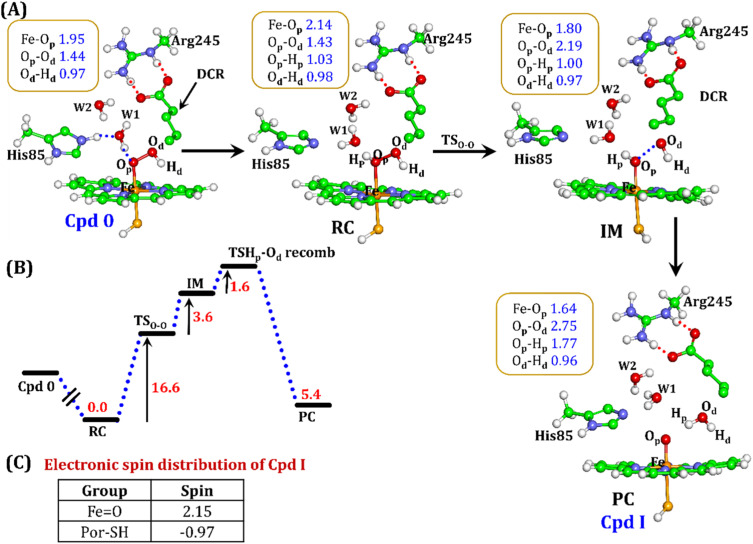
(A) QM/MM optimized geometries for the Cpd 0 → Cpd I formation. Note that His85 has been considered as the proton source. (B) Energy profile diagram for Cpd 0 → Cpd I formation. Energies are reported at the B2-D3 + ZPE level of theory. All energies are in kcal mol^−1^. (C) The spin distribution of the ultimate oxidant Cpd I.

To explore whether the conversion of Cpd 0 → Cpd I is initiated *via* a protonation from His85, we have performed QM/MM calculations. As can be seen in Fig. 10, the proton transfer from His85 to the heme moiety first generates Fe–H_2_O_2_ (RC), in a barrierless manner.

It is well documented in CYP450 s that Fe–H_2_O_2_ cleavage of proximal and distal oxygen (O_p_ and O_d_), either homolytic or heterolytic, generates the active oxidant Cpd I *via* dehydration.^54^ A PES scanning was performed to test a similar mechanistic pathway for Cpd I generation. However, in the present case, the O_p_–O_d_ cleavage occurred with a feasible energy barrier of 16.6 kcal mol^−1^, generating a free hydroxyl (O_d_–H_d_) at intermediate IM. The state IM was unstable on the potential energy surface, and recombination of proximal Hydrogen (Hp) to the free hydroxyl moiety generates a new water molecule (H_p_–O_d_–H_d_) and active oxidant Cpd I (PC in Fig. 10). Note that the generated Cpd I is 5.4 kcal mol^−1^ endothermic to RC (Fe–H_2_O_2_). Similarly, the Cpd 0 → Cpd I formation through Arg245 was also found to be energetically unfavorable (*cf.* Fig. S9†). Therefore, we deemed this pathway to be unfavourable.

#### 3.5.3 Pathway2: protonation *via* the substrate

**Fig. 11:**
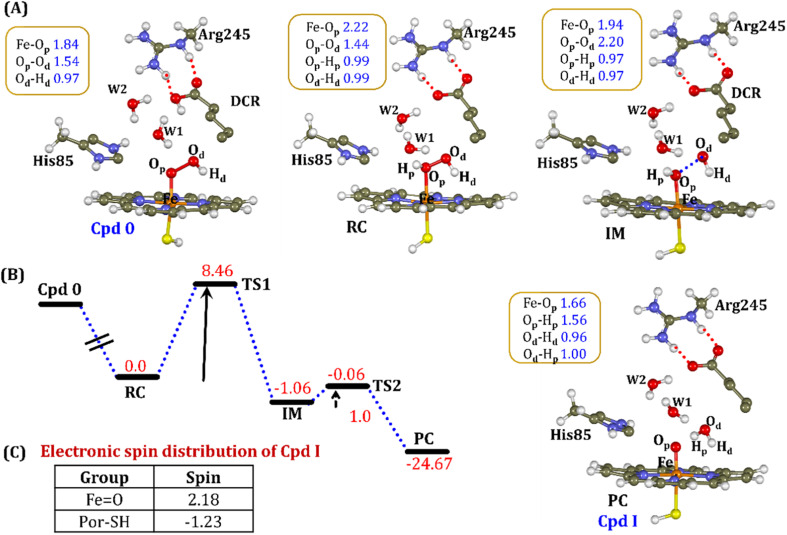
(A) QM/MM optimized geometries during the Cpd 0 → Cpd I formation. Note that the carboxylic proton of the substrate has been considered as the proton source. (B) Energy profile diagram for the Cpd 0 → Cpd I formation. Energies are reported at the B2-D3 + ZPE level of theory and relative to RC-opt (Fe–H_2_O_2_). All energies are in kcal mol^−1^. (C) The spin distribution of the ultimate oxidant Cpd I.

The second possibility of Cpd I generation involves the carboxylic acid hydrogen of the substrate, where the proton transfer from the substrate to the proximal oxygen (O_p_) occurred during geometry optimization only and generated Fe–H_2_O_2_ (RC). Further cleavage of the O_p_–O_d_ step occurred at an energy expense of 8.46 kcal mol^−1^ and generated the intermediate IM (*cf.*Fig. 11), along with a free hydroxyl group. It is noteworthy that here the IM is −1.06 kcal mol^−1^ lower than Fe–H_2_O_2_ and is stable on the potential energy surface. Further recombination of proximal hydrogen (H_p_) to the free hydroxyl (O_d_–H_d_) leads to the generation of a new water molecule (H_p_–O_d_–H_d_) and generates the energetically stable active oxidant Cpd I (*cf.*Fig. 11).

**Scheme 2:**
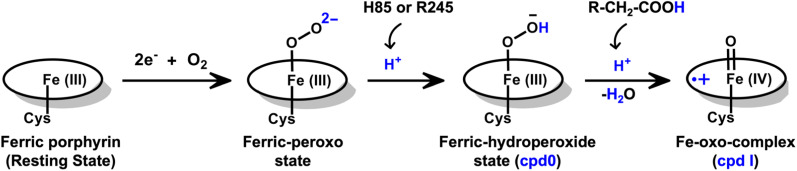
An overall reaction scheme for the formation of Cpd I species in the fused enzyme CYP450_OleT_-BM3R. The QM/MM calculations indicate that the initial protonation originates from either H85 or Arg245. Furthermore, the fatty acid substrate compensates for the absence of an acid–alcohol pair to facilitate the second protonation, ultimately leading to the formation of Cpd I within the fused CYP_OleT_-BM3R enzyme.

It is interesting to note that the Cpd I species, generated *via* this pathway, is highly stable (exothermic by −24.6 kcal mol^−1^) relative to RC. Thus, we can conclude that, in the fused protein OleT-BM3R, the Cpd 0 → Cpd I formation step is mediated by proton donation from the substrate. It is noteworthy that unlike other CYP450 monooxygenases, *e.g.*, CYP450_BM3_ where the hydrophobic terminal of the fatty acid substrate occupies the heme site, in the CYP450 peroxygenase family enzyme, the acidic terminal occupies the heme site. Therefore, this finding provides an evolutionary rationale that the present orientation of the substrate compensates for the lack of the acid–alcohol pair, which is needed for the second protonation, and thus the acid–alcohol pair is absent in CYP450 peroxygenases (Scheme 2).

## 4. Conclusion

The present study disentangles the mechanistic puzzles during the conversion of peroxide-based CYP450_OleT_ to a reductase-based monooxygenase. The work reveals a fascinating choreography of the enzyme, and its capability to sense and respond to external stimuli (here the fusion of reductase). Using extensive MD simulations, we showed here the manner in which a far-located (from catalytic center) Tyr359 residue transduces an interaction with Gly459 of the reductase domain into a signal that triggers the inflow of water and supplies the two protons needed for the reductase-based monooxygenase activity. These water chains further mediate the first protonation for the efficient formation of ferric-hydroperoxide (Cpd 0) necessary for the catalytic cycle. We demonstrate here that the acidic end of the fatty acid compensates for the lack of the acid–alcohol pair needed for the second protonation. This rationalizes the evolutionary mechanism whereby, unlike CYP450_BM3_, CYP450 peroxygenase positions the fatty acid substrates with its acidic terminal close to heme-porphyrin. The present study provides a broader perspective with two important take-home lessons:

• The regulation of the catalytic activity is not only limited to the first or second catalytic shell of an enzyme. In fact, far residing residues regulate the present catalytic mechanism.

• The positioning of tyrosine (Tyr359) at a similar position in other members of CYP450 peroxygenases can convert these enzymes to reductase-based monooxygenases.

## Data availability

Data necessary to produce the results are provided in the ESI.† It contains the QM optimized geometries for all species used in the calculations.

## Author contributions

SY performed the calculation. KDD supervised the project. SS and KDD wrote the manuscript.

## Conflicts of interest

There are no conflicts to declare.

## Supplementary Material

SC-015-D3SC06538C-s001
