# Supplementary material for: On the engineering of reductase-based-monooxygenase activity in CYP450 peroxygenases
Source: Chem Sci. 2024 Mar 7;15(14):5174–86. doi: 10.1039/d3sc06538c (PMC10988616; doi:10.1039/d3sc06538c)
Supplement: SC-015-D3SC06538C-s001 [file SC-015-D3SC06538C-s001.pdf]

Electronic Supplementary Material (ESI) for Chemical Science.
This journal is © The Royal Society of Chemistry 2024

Supporting Information

On the Engineering of Reductase-Based-Monooxygenase Activity in CYP450 Peroxygenases

Shalini Yadav, Sason Shaik* and Kshatresh Dutta Dubey*

1. Department of Chemistry, School of Natural Science, Shiv Nadar Institution of Eminence, NH91 Tehsil Dadri, Greater Noida, Uttar Pradesh 201314, India. Email: kshatresh.dubey@snu.edu.in

2. Institute of Chemistry, Edmond J. Safra Campus at Givat Ram, The Hebrew University, Jerusalem, 9190401 Israel. Email: sason@yfaat.ch.huji.ac.il

Table of Content:

Scheme S1: Scheme for the generic steps involve in Cpd I formation in CYP450 S2

Table S1: Trajectory Clustering Analysis S3

Figure S1: RMSD and RMSF Analysis of MD simulation at ferric peroxo state S3

Section S1: SPM Analysis S3

Figure S2: Pictorial representation of angle and distance change between two domains during MD simulation in ferric peroxo state S4

Table S2. Interaction energy calculated for the C-helix and α1-helix by MMPBSA calculations S4

Figure S3. Water occupancy and water flow near heme propionate in ferric peroxo state S5

Figure S4. Possible tunnels present at the axial site of the heme and alterations in B-C loop for water flow S5

Figure S5. QM/MM optimized QM zone for the dioxygen→ Cpd 0 formation through Arg66 S6

Figure S6. RMSD of OleT-BM3R in Cpd 0 state for 500 ns of simulation time S6

Figure S7. Water occupancy near 8 Å of heme in Cpd 0 state of OleT-BM3R S7

Figure S8. Disruption of salt bridge interaction between Arg66 and propionate in Cpd 0 state for 500 ns of simulation time. S7

Figure S9. QM/MM optimized geometries for the Cpd 0 →Cpd I formation via Arg245 S8

Coordinates of atoms in the QM region obtained from the QM/MM optimized geometries S8

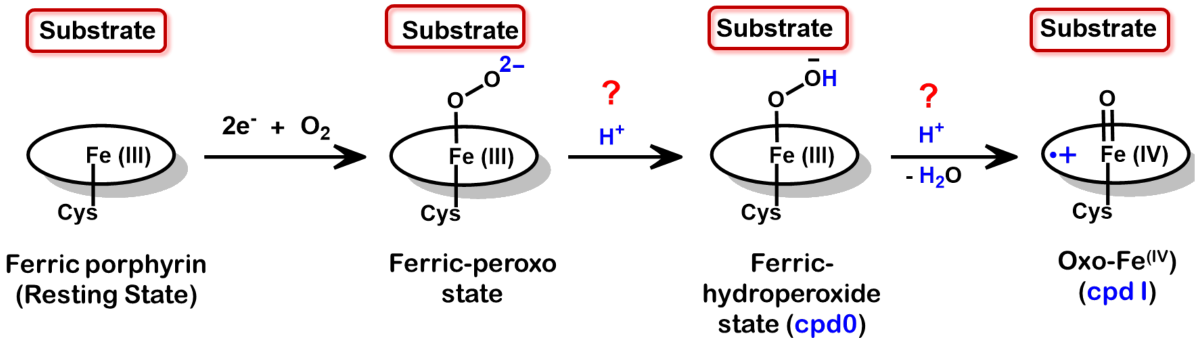

Scheme S1. Generic steps involved for the Cpd I formation in the monooxygenase activity in CYP450s. Consumption of two electrons (from the reductase partner) and one molecular oxygen converts the ferric porphyrin state to the ferric-peroxo state. Afterward, the subsequent protonation leads to the conversion of ferric-peroxo→Cpd 0 → Cpd I. It is important to note that the source of protons, i.e., acid-alcohol pair (in monooxygenases), is absent in CYP450 peroxygenases.

Table S1. Trajectory clustering analysis result of the 500 ns simulation of OleT-BM3R at ferric-peroxo heme state.

Cluster	Population
C1	41%
C2	37%
C3	12%
C4	6%
C5	5%

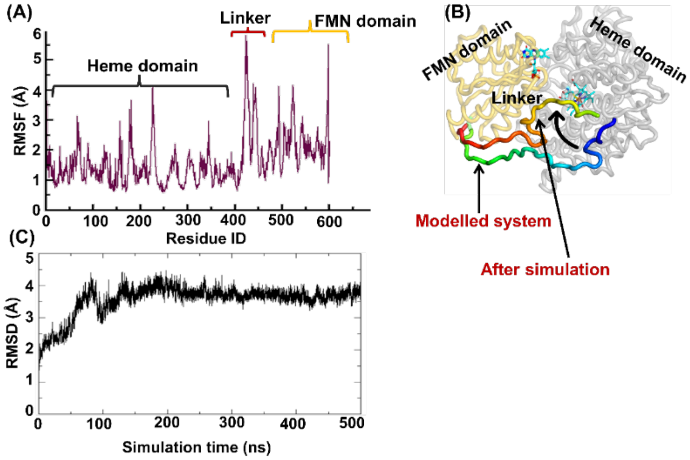

Figure S1. (A) Root mean square Fluctuation (RMSF) of whole complex (Heme domain +FMN domain) in peroxo-ferric state. (B) Pictorial representation of the most fluctuating region in OleT-BM3R. (B) Root mean square Deviation (RMSD) of whole complex (Heme domain +FMN domain) in peroxo-ferric state.

Section S1: SPM Analysis:

The SPM is a python program, as introduced by Romero-Rivera et al. in 2017 (reference 50). It **involves constructing a path based on calculated mean distances and correlation values**. Subsequently, it employs the Dijkstra algorithm within the igraph module to determine the shortest path lengths. The algorithm systematically goes all nodes in the graph, pinpointing the shortest path from the first to the last protein residue. This method reveals the graph's edges that are shorter, indicating higher correlation and centrality in the communication pathway. Only those edges with a significant contribution are depicted, and their representation is weighted in proportion to their contribution.

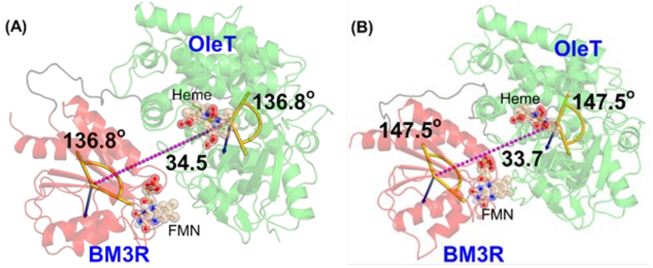

Figure S2: Variation in distance and angle between Heme binding domain (OleT) and reductase binding domain (BM3R) at the initial (A) and final (B) frame of MD simulation.

Table S2. Interaction energy calculated for the C-helix and α1-helix by MMPBSA calculations.

Energy Component	Energies (initial)	Energies (final)
BOND	157.6202	157.3773
ANGLE	433.7778	433.3059
DIHED	613.6804	598.3391
VDWAALS	-298.1813	-306.5617
EEL	-2875.4961	-2921.8208
1-4 VDW	175.423	174.3922
1-4 EEL	2179.453	2152.6855
EGB	-1473.999	-1408.4109
ESURF	34.7714	34.4181
G gas	386.277	287.7174
G solv	-1439.2276	-1373.9928
TOTAL	**-1052.9506**	**-1086.2754**
Difference	***33.32 kcal***	

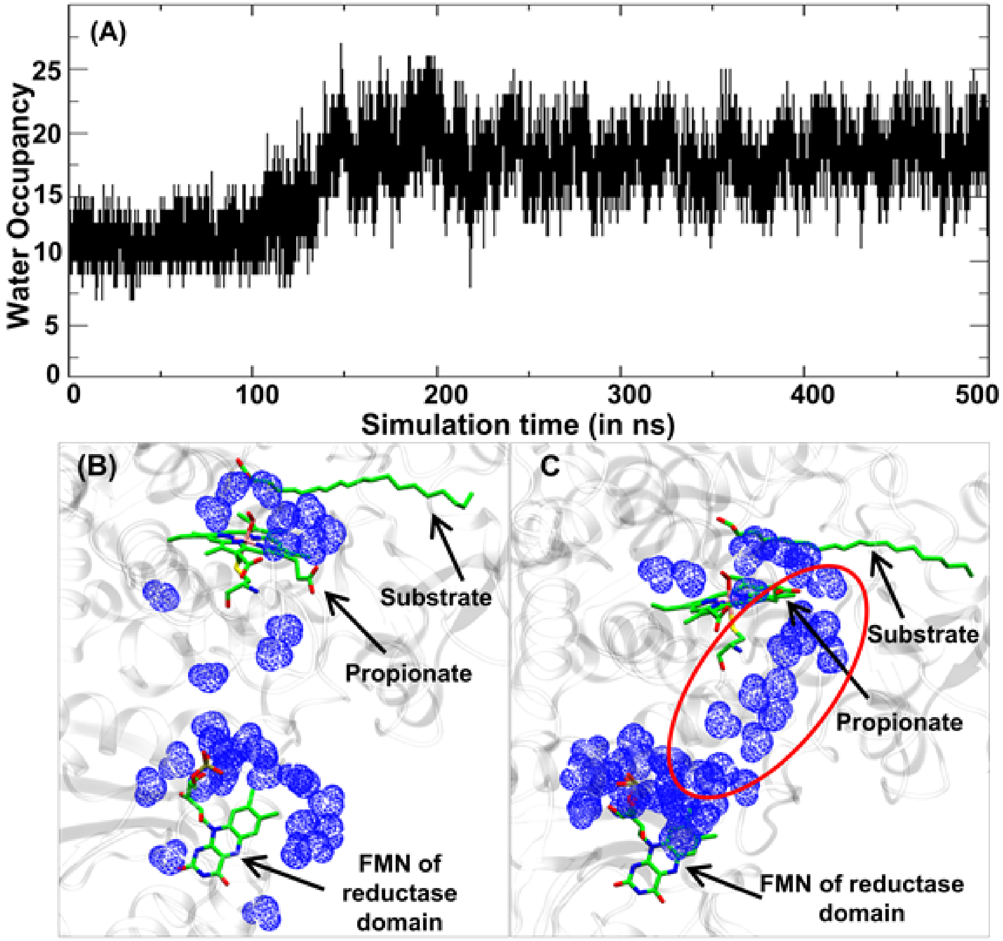

Figure S3. (A) Water occupancy near 5-8 Å range of heme. **(B,C)** Water population connecting the heme domain to the FMN binding reductase domain, at the initial (B) of MD simulation and after 200 ns (C) of MD simulation. The red highlighted part shows the connectivity of two domains via an organized water flow.

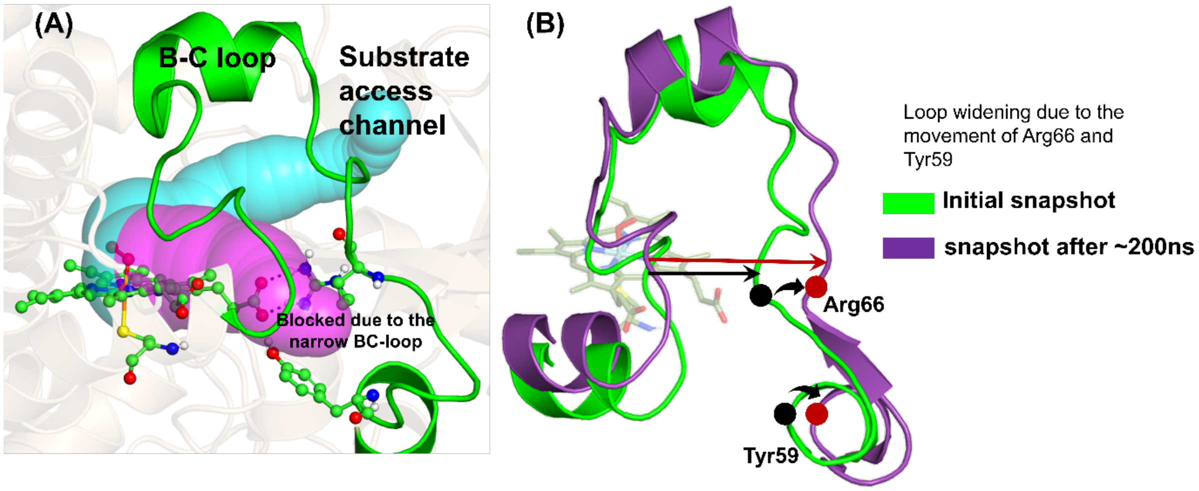

Figure S4. (A) Possible tunnels present at the axial site of the heme. Note that tunnels present at the propionate side is blocked due to the narrowness of BC-loop. **(B)** Widening of B-C loop due to the movement of Arg66 and Tyr59 residue near propionate.

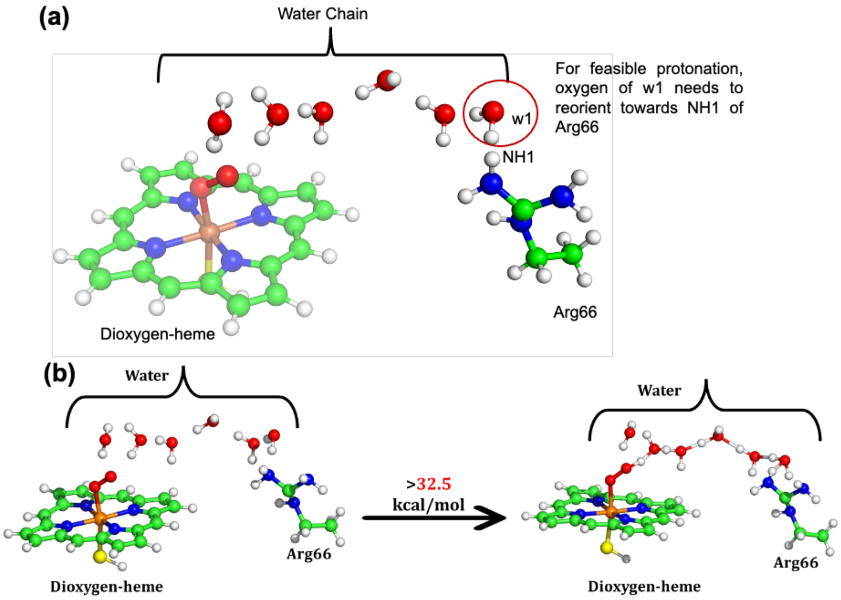

Figure S5. (a) Graphical representation of unfavorable protonation from Arg66. (b) Geometries obtained during potential energy surface scanning for the dioxygen→ Cpd 0 formation through Arg66. Note that the reorientation of water w1 is energetically demanding which makes the protonation from Arg66 pathway unfavorable.

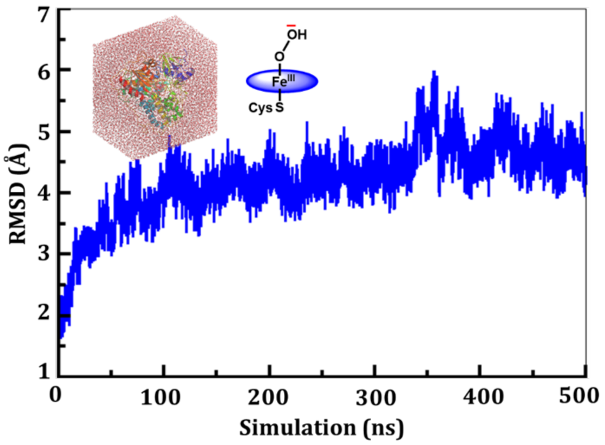

Figure S6. RMSD of OleT-BM3R in Cpd 0 state for 500 ns of simulation time.

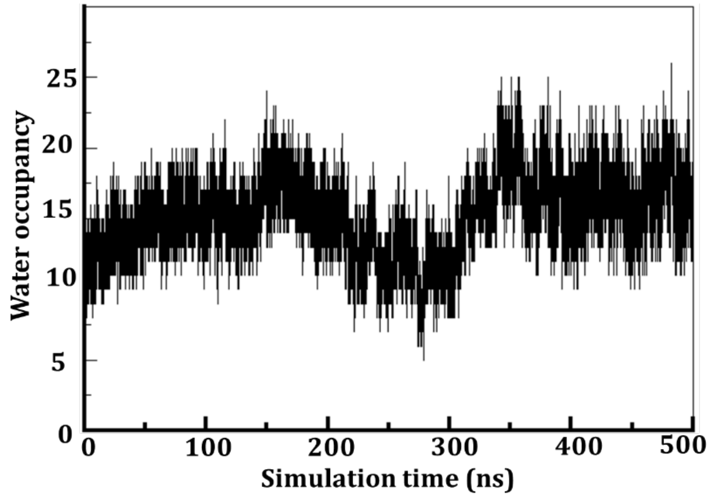

Figure S7. Water occupancy near 8 Å of heme in cpd 0 state of OleT-BM3R.

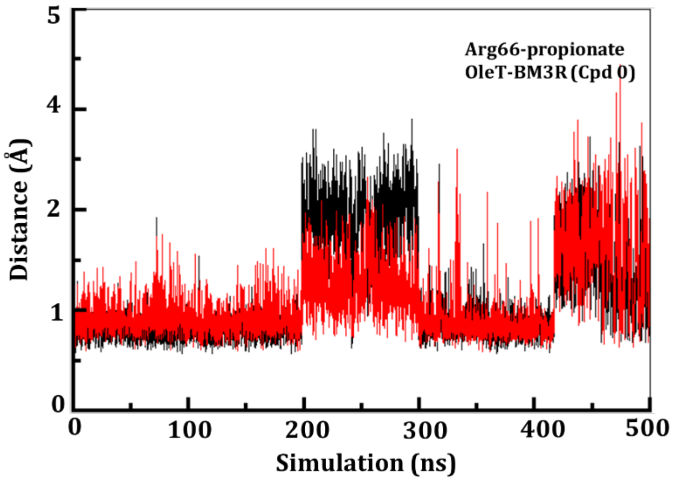

Figure S8. Disruption of salt bridge interaction between Arg66 and propionate in Cpd 0 state for 500 ns of simulation time.

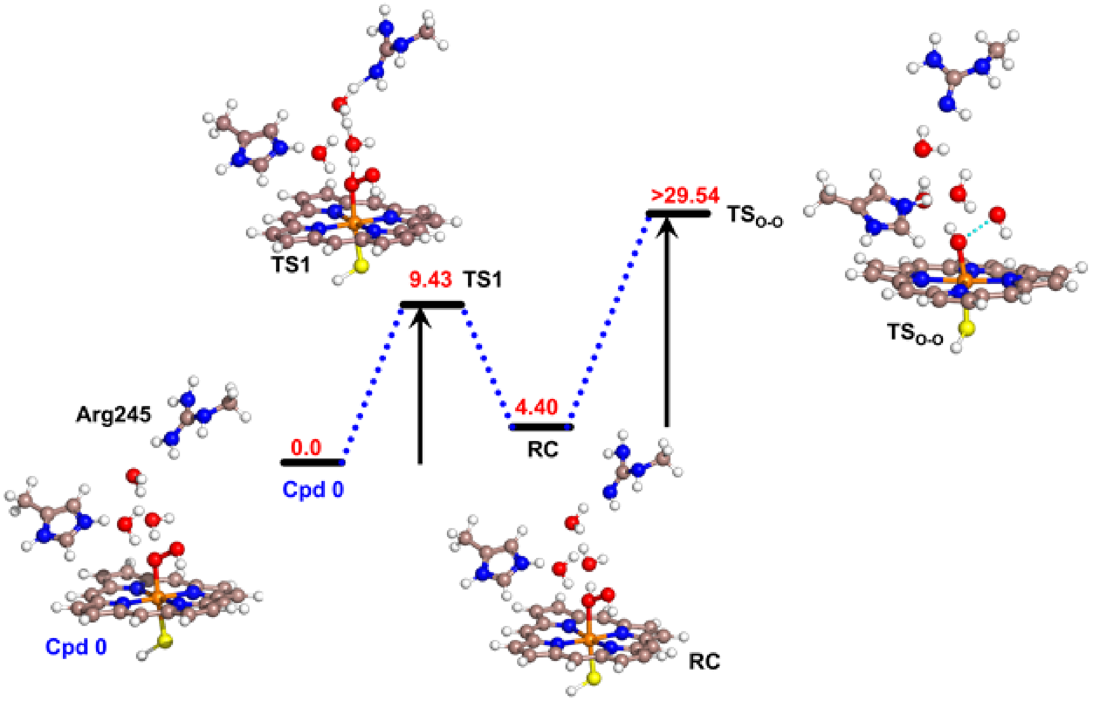

Figure S9. QM/MM optimized geometries for the Cpd 0 → Cpd I formation. Note that here Arg245 have been considered as the proton source. Energy profile diagram for the Cpd 0 → Cpd I formation. All energies are in kcal/mol.

Coordinates of atoms in the QM region obtained from the QM/MM optimized geometries.

Fe-OO^{2-} to FeOOH^{-} (Cpd 0 formation) via His85

RC

C	58.0158903	63.7641262	52.3023197
H	57.8454601	63.9697284	51.2312206
H	59.0358365	64.1170625	52.5265732
C	57.9813438	62.2722514	52.4883341
N	58.7191574	61.5765184	53.4393596
H	59.1901458	62.0090499	54.2399625
C	58.6808949	60.2462802	53.2031084
H	59.2216088	59.3518374	53.7236758
N	57.9131420	60.0846235	52.1178412
H	57.7964926	59.1209871	51.6455390
C	57.4677504	61.3020502	51.6634343
H	56.8618206	61.3943627	50.7634925
S	61.0526657	56.2455772	58.3092046
N	59.4486955	54.9048676	56.1947365
C	59.8793300	53.6113309	56.2133833
C	58.1256820	54.8699026	56.5170722
C	58.7774817	52.7002623	56.5472018
C	57.6612522	53.4809446	56.7222302

C	57.3481264	56.0238183	56.6752945
H	56.2925661	55.8886498	56.9093728
C	57.7672182	57.3600408	56.6449539
N	59.0328838	57.7912428	56.3625814
C	59.0299351	59.1520976	56.4693887
C	57.7046847	59.6013390	56.8743788
C	56.9038971	58.4924028	56.9726994
C	60.1470140	59.9716066	56.2657539
H	60.0143975	61.0443335	56.4270025
C	61.4410274	59.5535101	55.9300107
C	62.6011749	60.4192967	55.7426062
C	63.1471730	58.2430833	55.5143177
N	61.8079027	58.2497419	55.7799174
C	63.6814480	59.6002619	55.4838226
C	63.9251103	57.0981740	55.3194722
H	64.9910900	57.2762979	55.1696618
C	63.5025012	55.7711098	55.3068264
C	64.3680631	54.6149070	55.0839244
N	62.2162087	55.3644731	55.5004964
C	62.2313283	53.9990849	55.4462366
C	61.1710993	53.2032782	55.8792391
H	61.3977591	52.1492863	56.0042021
Fe	60.6043281	56.6081438	55.9381642
C	63.5697544	53.4996241	55.0971408
O	60.1796306	56.6834203	54.1378078
O	59.6875686	57.8639218	53.5222888
O	57.7830223	57.6817139	51.0709280
H	57.6396605	57.3583468	52.0068483
H	58.7493458	57.5023421	50.8864814
H	57.3056417	64.3741556	52.8604221
H	61.3286482	57.4292020	58.9628201
H	58.8607238	51.6148640	56.6024149
H	56.6302847	53.2212497	56.9625046
H	57.4255983	60.6052780	57.1941981
H	55.8428896	58.4782257	57.2219566
H	62.6266237	61.5049789	55.8359532
H	64.7289700	59.8595447	55.3304043
H	65.4474456	54.6151616	54.9322647
H	63.9416413	52.5069628	54.8433873

PC

C	57.9288311	64.0119023	52.1726224
H	57.7674399	64.2888775	51.1155774
H	58.9668324	64.2844368	52.4217118
C	57.7417248	62.5321546	52.2942764
N	58.3856707	61.7436126	53.2197096
H	58.9639698	62.0930770	53.9873618
C	58.0504099	60.4473746	52.9800720
H	58.4730643	59.6126874	53.5451498
N	57.2155850	60.3620791	51.9595359
H	57.2480639	58.6280537	51.2892884
C	57.0136897	61.6440254	51.5260570
H	56.3795141	61.8568002	50.6637088
S	61.0172365	56.2227908	58.3648116
N	59.5325335	54.8364600	56.2575562
C	59.9433039	53.5364474	56.2250567
C	58.2132476	54.8127757	56.6093189
C	58.8354793	52.6344033	56.5592627
C	57.7359686	53.4254455	56.7857325
C	57.4460385	55.9685942	56.7927856
H	56.3973074	55.8354931	57.0547080
C	57.8579940	57.3052512	56.7148636
N	59.1175827	57.7283501	56.3826348

C	59.1114377	59.0943045	56.4282096
C	57.7934159	59.5593772	56.8348709
C	57.0018783	58.4491611	57.0086371
C	60.2291787	59.9007518	56.1877493
H	60.0863075	60.9803450	56.2694500
C	61.5379570	59.4678610	55.9551096
C	62.7100650	60.3318072	55.8418585
C	63.2788970	58.1476419	55.7869546
N	61.9180342	58.1581146	55.9010337
C	63.8133307	59.5055282	55.7589525
C	64.0658099	56.9945662	55.7060921
H	65.1452515	57.1496901	55.6644757
C	63.6245791	55.6758607	55.6335807
C	64.4879750	54.5153211	55.4338399
N	62.3201891	55.2844923	55.6828088
C	62.3158672	53.9247976	55.5314715
C	61.2264199	53.1213648	55.8627634
H	61.4259422	52.0557374	55.9076809
Fe	60.7110970	56.5151035	56.0558434
C	63.6718883	53.4262511	55.2629834
O	60.2674024	56.5051326	54.1959280
O	60.4958380	57.7510988	53.4501449
O	57.4757241	57.6951700	51.1011473
H	57.5244185	57.3282341	52.0233868
H	59.9104833	57.5843833	52.6919483
H	57.2419805	64.6020081	52.7793172
H	61.3041983	57.4112373	59.0048077
H	58.9072743	51.5474548	56.5974169
H	56.7067846	53.1718567	57.0397799
H	57.5237915	60.5725853	57.1328295
H	55.9494560	58.4475412	57.2923124
H	62.7035171	61.4213828	55.8709992
H	64.8770858	59.7419290	55.7343174
H	65.5769132	54.5057668	55.4806311
H	64.0443052	52.4517419	54.9472231

Cpd 0 formation Pathway via Arg245

RC

C	58.1765507	52.9619535	47.1586218
H	59.0670665	52.9578464	47.7889875
H	58.1548215	52.0321799	46.5912121
C	58.2827141	54.1403489	46.1810661
H	59.1142652	53.9384800	45.5048584
H	57.3693666	54.2140949	45.5894716
N	58.5580349	55.3964178	46.8991391
H	59.4794496	55.4911904	47.3396942
C	57.7051729	56.3050911	47.3253905
N	56.4673248	56.3767356	46.9304169
H	56.1284632	55.7419676	46.2373426
H	55.8982543	57.1070526	47.3323913
N	58.0779028	57.1723315	48.2020714
H	59.0229800	57.0960819	48.5982253
H	57.4046990	57.7413835	48.6960542
S	61.0526657	56.2455772	58.3092046
N	59.4486955	54.9048676	56.1947365
C	59.8793300	53.6113309	56.2133833
C	58.1256820	54.8699026	56.5170722
C	58.7774817	52.7002623	56.5472018
C	57.6612522	53.4809446	56.7222302
C	57.3481264	56.0238183	56.6752945
H	56.2925661	55.8886498	56.9093728
C	57.7672182	57.3600408	56.6449539
N	59.0328838	57.7912428	56.3625814

C	59.0299351	59.1520976	56.4693887
C	57.7046847	59.6013390	56.8743788
C	56.9038971	58.4924028	56.9726994
C	60.1470140	59.9716066	56.2657539
H	60.0143975	61.0443335	56.4270025
C	61.4410274	59.5535101	55.9300107
C	62.6011749	60.4192967	55.7426062
C	63.1471730	58.2430833	55.5143177
N	61.8079027	58.2497419	55.7799174
C	63.6814480	59.6002619	55.4838226
C	63.9251103	57.0981740	55.3194722
H	64.9910900	57.2762979	55.1696618
C	63.5025012	55.7711098	55.3068264
C	64.3680631	54.6149070	55.0839244
N	62.2162087	55.3644731	55.5004964
C	62.2313283	53.9990849	55.4462366
C	61.1710993	53.2032782	55.8792391
H	61.3977591	52.1492863	56.0042021
Fe	60.6043281	56.6081438	55.9381642
C	63.5697544	53.4996241	55.0971408
O	60.1796306	56.6834203	54.1378078
O	59.6875686	57.8639218	53.5222888
O	57.7830223	57.6817139	51.0709280
H	57.6396605	57.3583468	52.0068483
H	58.7493458	57.5023421	50.8864814
H	57.2794817	53.0204952	47.7749879
H	61.3286482	57.4292020	58.9628201
H	58.8607238	51.6148640	56.6024149
H	56.6302847	53.2212497	56.9625046
H	57.4255983	60.6052780	57.1941981
H	55.8428896	58.4782257	57.2219566
H	62.6266237	61.5049789	55.8359532
H	64.7289700	59.8595447	55.3304043
H	65.4474456	54.6151616	54.9322647
H	63.9416413	52.5069628	54.8433873

PC

C	58.4509957	53.2672326	47.3007309
H	59.3163708	53.2123358	47.9809129
H	58.4593740	52.3487351	46.6875195
C	58.6304655	54.4915433	46.3822234
H	59.4436087	54.2392717	45.6799780
H	57.7319447	54.6443211	45.7576615
N	59.0073669	55.7397187	47.0304805
H	59.9410147	55.7037593	47.4777483
C	58.1365545	56.5386101	47.7495688
N	56.7983916	56.5105619	47.3471205
H	56.6390358	56.3896584	46.3518183
H	56.2991032	57.3098763	47.7405900
N	58.4663934	57.2912210	48.7419257
H	59.4633410	57.2075734	49.0049216
H	57.7945646	57.3365631	50.3445301
S	60.9949659	56.1921448	58.4431162
N	59.4192060	54.7294533	56.4290144
C	59.8300907	53.4317880	56.3850626
C	58.0964066	54.7040751	56.7616698
C	58.7121163	52.5241042	56.6684492
C	57.6119959	53.3131694	56.8961739
C	57.3360321	55.8571613	56.9815562
H	56.2995807	55.7150089	57.2833802
C	57.7373502	57.1977738	56.9034945
N	58.9769637	57.6326844	56.5144736
C	58.9664075	58.9994190	56.5838316

C	57.6618124	59.4522822	57.0393552
C	56.8847883	58.3355225	57.2391399
C	60.0719100	59.8229095	56.3217235
H	59.9282148	60.9008252	56.4311257
C	61.3603230	59.3984799	55.9847156
C	62.5203292	60.2636831	55.7914150
C	63.0771807	58.0808867	55.6410603
N	61.7303361	58.0881878	55.8700547
C	63.6064351	59.4396414	55.5808395
C	63.8681254	56.9343363	55.5286971
H	64.9363136	57.1107826	55.3979347
C	63.4555719	55.6045874	55.5607370
C	64.3349562	54.4521476	55.3736190
N	62.1688791	55.1886583	55.7267521
C	62.1912109	53.8226403	55.6621769
C	61.1243610	53.0199634	56.0584859
H	61.3430462	51.9622372	56.1663717
Fe	60.5543323	56.4164640	56.1274523
C	63.5407288	53.3354595	55.3413020
O	60.0942601	56.2704822	54.3008793
O	59.6192882	57.4492354	53.5871799
O	57.6141771	57.4474263	51.3196585
H	57.0100350	56.7544045	51.6294879
H	59.1842721	57.0497987	52.8079057
H	57.5249297	53.2624964	47.8755756
H	61.2761660	57.3964490	59.0554275
H	58.7746692	51.4365205	56.6321741
H	56.5760257	53.0574460	57.1185212
H	57.3845743	60.4670099	57.3248968
H	55.8398332	58.3400951	57.5491643
H	62.5420859	61.3501918	55.8756326
H	64.6569034	59.6964467	55.4443315
H	65.4231041	54.4597852	55.3108270
H	63.9211340	52.3513727	55.0675644

Cpd 0 formation via Arg66

RC

C	62.7486645	69.3617440	57.1121149
H	62.4789127	70.0354693	56.2806275
H	63.8152304	69.1062118	57.0113086
C	61.8974378	68.0979400	56.9651007
H	62.1763618	67.3665920	57.7410841
H	60.8361912	68.3493689	57.1340100
N	62.0525132	67.4676928	55.6459051
H	62.4107193	66.5109998	55.7304508
C	60.9763933	67.4846089	54.7435059
N	60.6761666	68.7299281	54.0644912
H	60.7856356	69.5229447	54.7056521
H	59.6771466	68.7341782	53.8361874
N	60.9809176	66.3634246	53.8447827
H	60.0668026	66.3083997	53.3813484
H	61.0890937	65.5108720	54.4094288
S	60.9651893	56.2001586	58.4173130
N	59.4110121	54.7756203	56.3604397
C	59.8240404	53.4750000	56.3467361
C	58.0748428	54.7521961	56.6476098
C	58.7037278	52.5734267	56.6256822
C	57.5949513	53.3646294	56.8066705
C	57.2982674	55.9078334	56.7849280
H	56.2413255	55.7749859	57.0110417
C	57.7129815	57.2444273	56.7332908
N	58.9860647	57.6685693	56.4569649
C	58.9865936	59.0339155	56.5702239

C	57.6628763	59.4925989	56.9569205
C	56.8560345	58.3839439	57.0455293
C	60.1049989	59.8532706	56.3750641
H	59.9692504	60.9281667	56.5114548
C	61.3921978	59.4217210	56.0530650
C	62.5543087	60.2833145	55.8783583
C	63.1001851	58.1051502	55.6600286
N	61.7537289	58.1086547	55.9028478
C	63.6317361	59.4628035	55.6287282
C	63.8836771	56.9621888	55.4990442
H	64.9495856	57.1394631	55.3555498
C	63.4598545	55.6361513	55.5048932
C	64.3231842	54.4829815	55.2842175
N	62.1692927	55.2279690	55.6895933
C	62.1846980	53.8574987	55.6228902
C	61.1179612	53.0593791	56.0275581
H	61.3372592	52.0031239	56.1465761
Fe	60.5800532	56.4509050	56.1658149
C	63.5233482	53.3676156	55.2761012
O	60.0433521	56.3823169	54.2149993
O	59.6704127	57.4549735	53.5786132
O	63.0607180	66.2653056	51.9456828
H	63.7729087	65.6898519	52.2896402
H	62.3740301	66.2667444	52.6701361
O	61.1944328	58.9421184	51.7817161
H	60.8339536	58.4406259	52.5458865
H	60.9910886	58.3416382	51.0090242
O	63.5375959	60.1206075	52.3917313
H	63.5088876	60.0515955	53.3635578
H	62.7235651	59.6273396	52.0990112
O	65.2474987	64.4642441	52.8241261
H	64.9589692	63.6594844	52.3148774
H	64.6405854	64.4769088	53.6284607
O	58.2483599	57.6468547	51.1894335
H	58.5913164	57.3358534	52.0637938
H	59.0896460	57.6128386	50.6642271
O	64.3420949	62.3913279	51.3331524
H	64.0997007	61.5084655	51.7589774
H	63.4793773	62.7081926	51.0303018
H	62.6278129	69.8827021	58.0618858
H	61.2531021	57.3914435	59.0515793
H	58.7689885	51.4854026	56.6222258
H	56.5569139	53.1096045	57.0199957
H	57.3983229	60.4983713	57.2832645
H	55.7942434	58.3853722	57.2918312
H	62.5832098	61.3661028	56.0000192
H	64.6748901	59.7300214	55.4599339
H	65.4056796	54.4906915	55.1568934
H	63.8960477	52.3803374	55.0032569

TS

C	62.7841288	69.3261067	57.0545091
H	62.5414009	70.0005622	56.2152991
H	63.8489524	69.0532605	56.9797810
C	61.9141604	68.0722368	56.8959985
H	62.1362855	67.3652008	57.7110282
H	60.8527542	68.3538163	57.0026403
N	62.1023888	67.3669473	55.6075917
H	62.4006614	66.4018721	55.8043641
C	60.9881386	67.2634031	54.7140867
N	60.5698576	68.4759111	54.0518264
H	60.5593824	69.2802065	54.6889341
H	59.5993328	68.3176047	53.7598564

N	60.9740016	66.1451841	53.8467780
H	61.8303855	66.1874933	53.1994820
H	61.0413746	65.2944072	54.4113745
S	60.9502445	56.2024010	58.4114304
N	59.4233908	54.7867049	56.3454373
C	59.8317548	53.4831999	56.3277756
C	58.0856806	54.7625997	56.6359576
C	58.7120112	52.5842185	56.6082940
C	57.6045608	53.3768929	56.7930212
C	57.3089136	55.9162967	56.7755792
H	56.2512143	55.7839828	56.9988383
C	57.7208687	57.2516918	56.7226351
N	58.9980108	57.6774855	56.4516495
C	58.9927427	59.0442100	56.5616968
C	57.6669334	59.5007282	56.9401101
C	56.8623163	58.3894665	57.0272262
C	60.1167203	59.8598482	56.3831093
H	59.9826522	60.9341598	56.5184684
C	61.4075576	59.4251692	56.0898894
C	62.5771299	60.2824374	55.9563007
C	63.1174452	58.1087890	55.7005535
N	61.7649278	58.1094797	55.9233918
C	63.6512180	59.4641616	55.7046779
C	63.8989420	56.9693497	55.5263522
H	64.9655224	57.1467677	55.3908657
C	63.4706031	55.6447651	55.5081625
C	64.3288312	54.4922954	55.2775284
N	62.1763269	55.2402007	55.6834787
C	62.1900808	53.8667297	55.6059355
C	61.1238376	53.0666853	56.0034213
H	61.3427298	52.0092191	56.1151756

Fe	60.6049850	56.4607918	56.1829389
C	63.5269248	53.3782095	55.2590181
O	59.8754776	56.4706340	54.1872487
O	59.6932764	57.4852845	53.3779869
O	63.3197171	66.6379066	52.5911006
H	63.8034486	65.7579064	52.5010989
H	63.4484244	66.8964542	53.5235415
O	61.1569472	58.8741775	52.2004163
H	60.5930167	58.1840678	52.9772537
H	61.0690020	58.3845040	51.3434180
O	63.3371857	60.0795229	52.3900681
H	63.4509054	60.1096530	53.3516941
H	62.1602466	59.4001639	52.2818833
O	64.7752881	64.4220902	52.5600228
H	64.5040171	63.5286696	52.1409788
H	64.4844360	64.3752671	53.5241393
O	58.1894511	57.9411636	51.0762708
H	58.3742640	57.5219046	51.9442308
H	59.0950535	57.8493742	50.6611516
O	64.0814378	62.2068856	51.3772517
H	63.8050880	61.2452203	51.8544064
H	63.2360569	62.4303276	50.9606376
H	62.6552781	69.8501820	58.0015095
H	61.2423615	57.3937988	59.0435587
H	58.7755969	51.4960908	56.6097174
H	56.5675234	53.1215618	57.0107978
H	57.3989572	60.5022221	57.2766601
H	55.8001689	58.3878388	57.2719864
H	62.6171510	61.3604031	56.1126824
H	64.6932618	59.7328857	55.5314746
H	65.4115041	54.4968604	55.1515695

H 63.9007071 52.3920244 54.9837130

Cpd 0 to Cpd I formation via His85

Cpd 0

C	46.5099010	56.6465712	39.2488308
H	45.7268775	56.4667960	38.4962771
H	47.2394040	57.3286511	38.7705314
C	47.2397223	55.3592699	39.4755545
N	48.4544017	55.2858833	40.1461085
H	48.9252562	56.0510113	40.6556566
C	48.9757306	54.0614377	39.9809615
H	49.9110989	53.7010293	40.4044647
N	48.1498091	53.3452392	39.2300431
H	48.4196664	52.3495067	38.8698645
C	47.0606688	54.1196667	38.9056029
H	46.2798321	53.7303957	38.2581877
C	45.8404196	46.9700339	32.4589503
H	46.5184458	46.7914199	31.6078666
H	44.8246387	47.0781160	32.0570813
N	46.2714427	48.1364470	33.2027379
H	47.1809654	48.0117893	33.7397152
C	45.4544917	49.0458626	33.7543266
N	44.1850599	49.2240052	33.3159408
H	43.9578926	48.9968907	32.3546415
H	43.6923341	50.0168359	33.7248482
N	45.8976522	49.7678779	34.7728687
H	46.8281113	49.5930845	35.2159616
H	45.3439879	50.5431664	35.1345112
S	49.1256541	48.4780223	44.4894805
N	47.8204596	47.4796124	42.1915260
C	48.1657304	46.1578211	42.1599888
C	46.4674783	47.5183205	42.4094350
C	46.9927598	45.3235241	42.3929007
C	45.9180161	46.1655604	42.5553252
C	45.7337061	48.7060769	42.4862027
H	44.6512039	48.6338526	42.5784968
C	46.2320384	50.0090018	42.5024265
N	47.5594578	50.3474692	42.5083964
C	47.6147639	51.7041164	42.6816078
C	46.2729467	52.2563642	42.7395025
C	45.4048184	51.1996548	42.6346687
C	48.7996674	52.4351682	42.7925888
H	48.7159802	53.4831209	43.0710713
C	50.0849462	51.9359795	42.5853710
C	51.2951849	52.7400277	42.5511686
C	51.7419320	50.5615134	42.1962957
N	50.3818599	50.6232847	42.3380348
C	52.3354565	51.8825552	42.3274045
C	52.4750193	49.4081099	41.9346187
H	53.5560001	49.5345227	41.8780551
C	51.9743520	48.1198568	41.7418407
C	52.7747460	46.9628710	41.3973215
N	50.6525611	47.7707394	41.8202985
C	50.6001267	46.4015223	41.6269907
C	49.4459272	45.6656566	41.8935273
H	49.5640973	44.5918356	41.9649847
C	51.9210521	45.8843216	41.2715260
Fe	49.0893647	49.0622436	42.2409916
O	48.8353102	49.4367676	40.3441342
H	49.9032098	47.9108111	40.0521528
O	49.2677099	48.3665277	39.4679123
O	48.3940461	49.4788402	35.9674515
C	48.8908857	48.3747904	35.5868723

O	48.4368072	47.6726524	34.6477888
C	50.1375846	47.8482292	36.3321927
C	51.2310878	48.9164752	36.5471491
C	52.3770193	48.5374104	37.5226353
C	53.4576495	49.6355840	37.6888144
O	46.4491431	51.4804534	37.0582185
H	47.2425349	51.0883987	36.6582398
H	46.3739849	50.9294639	37.8944960
O	49.2470312	51.1197439	38.4840240
H	49.1860063	50.5279568	39.3226195
H	48.9174257	50.5135311	37.7920238
H	46.0528404	57.1385843	40.1073679
H	45.8655839	46.0694911	33.0725108
H	49.6891562	49.4856321	45.2454800
H	46.9952537	44.2409468	42.2660652
H	44.8717265	45.9659184	42.7866003
H	46.0272752	53.3119376	42.8555944
H	44.3180935	51.2296727	42.5559599
H	51.3607781	53.8241252	42.6433490
H	53.3930441	52.1250812	42.2236900
H	53.8604444	46.9932076	41.3056380
H	52.2490820	44.9094990	40.9107152
H	49.7800594	47.5005404	37.3013962
H	50.5326044	46.9775704	35.8087618
H	51.6517201	49.1957235	35.5811491
H	50.7487118	49.8179451	36.9249488
H	51.9393685	48.3074465	38.4940495
H	52.8514380	47.6127052	37.1941319
H	54.2645382	49.2856377	38.3326587
H	53.9111730	49.8708381	36.7259857
H	53.0777202	50.5625382	38.1183220

TS1

C	46.5134641	56.6029077	39.2669113
H	45.7317047	56.4054730	38.5171696
H	47.2373316	57.2829774	38.7763450
C	47.2555204	55.3244292	39.5131925
N	48.4794719	55.2808558	40.1668423
H	48.9474444	56.0598617	40.6548297
C	49.0092804	54.0539589	40.0076114
H	49.9575850	53.7204739	40.4232698
N	48.1903646	53.3044152	39.2867092
H	48.5605989	52.1850094	38.9250885
C	47.0907809	54.0677962	38.9697196
H	46.3049338	53.6679873	38.3345939
C	45.8665616	46.9707528	32.4809679
H	46.5558272	46.7960632	31.6381265
H	44.8567020	47.0852352	32.0653871
N	46.2945700	48.1282247	33.2408426
H	47.1903928	47.9899857	33.7960669
C	45.4751468	49.0413847	33.7833840
N	44.2156878	49.2317513	33.3202920
H	44.0109024	49.0201208	32.3503310
H	43.7212902	50.0266610	33.7236308
N	45.9050119	49.7521497	34.8149432
H	46.8273739	49.5701693	35.2718453
H	45.3624173	50.5405497	35.1638962
S	49.1278299	48.5002577	44.4488595
N	47.7985121	47.5059196	42.1786519
C	48.1424185	46.1830755	42.1524878
C	46.4458705	47.5475172	42.4013763
C	46.9702374	45.3516014	42.3968577
C	45.8964340	46.1959664	42.5573594

C	45.7135306	48.7349921	42.4696980
H	44.6308286	48.6644701	42.5582520
C	46.2146777	50.0359942	42.4809908
N	47.5430788	50.3735591	42.4950341
C	47.5985657	51.7325410	42.6601488
C	46.2582287	52.2852790	42.7089450
C	45.3895019	51.2284762	42.6024918
C	48.7814353	52.4628564	42.7698437
H	48.6986256	53.5140090	43.0325908
C	50.0623284	51.9600986	42.5618707
C	51.2747749	52.7591650	42.5369633
C	51.7151881	50.5834233	42.1555032
N	50.3544009	50.6467109	42.2989038
C	52.3123585	51.9007848	42.3048878
C	52.4448947	49.4339965	41.8750572
H	53.5250473	49.5590839	41.8093800
C	51.9403333	48.1495076	41.6795517
C	52.7352922	46.9931165	41.3276490
N	50.6172245	47.8011684	41.7697423
C	50.5639909	46.4287631	41.5824756
C	49.4184513	45.6906087	41.8727289
H	49.5428006	44.6180544	41.9522001
C	51.8800138	45.9137142	41.2130264
Fe	49.0677310	49.0855718	42.2391252
O	48.7524320	49.4352982	40.3274042
H	49.8243929	47.9161223	40.0355496
O	49.1915963	48.3761416	39.4492866
O	48.3772742	49.4646529	36.0306820
C	48.8752760	48.3563066	35.6667881
O	48.4198036	47.6368180	34.7418026
C	50.1198267	47.8397919	36.4231017
C	51.1594937	48.9343847	36.7367604
C	52.2425582	48.5798974	37.7890046
C	53.3311492	49.6688676	37.9603305
O	46.5408472	51.4662382	37.0525274
H	47.3085115	51.0268590	36.6512165
H	46.4141417	50.9162806	37.8815357
O	49.2166392	51.1366578	38.6746964
H	49.0835901	50.4487723	39.5322860
H	48.8460772	50.5981004	37.9513689
H	46.0541324	57.1071096	40.1171242
H	45.8778117	46.0651631	33.0874725
H	49.6889618	49.5138117	45.1986507
H	46.9725678	44.2677274	42.2816234
H	44.8504581	45.9990448	42.7923558
H	46.0128019	53.3397694	42.8349718
H	44.3032331	51.2582542	42.5176354
H	51.3448219	53.8418801	42.6413113
H	53.3710190	52.1411548	42.2072796
H	53.8210354	47.0193672	41.2352386
H	52.2048450	44.9376116	40.8527827
H	49.7417186	47.4154658	37.3531816
H	50.5664089	47.0161775	35.8660642
H	51.6379497	49.2406611	35.8065229
H	50.6270783	49.8169032	37.0913901
H	51.7489612	48.4029097	38.7445696
H	52.7182187	47.6335650	37.5315765
H	54.0674545	49.3584966	38.7016748
H	53.8718189	49.8126314	37.0248759
H	52.9439604	50.6372665	38.2771198

RC

C	46.4560213	56.4774987	39.3768866

H	45.7649635	55.6591746	39.1428459
H	46.4830556	57.0954769	38.4562423
C	47.7758359	55.8063913	39.5843277
N	48.9697633	56.3788883	39.9694367
H	49.1452416	57.2938878	40.3992383
C	49.9311695	55.4215234	39.8906279
H	50.9617578	55.6193194	40.1807500
N	49.4400413	54.2742602	39.4665179
H	49.7735939	52.4754113	39.2645108
C	48.0998679	54.4993767	39.2764278
H	47.4395812	53.7194787	38.8972485
C	45.8191747	46.9338837	32.4495352
H	46.4864817	46.7480854	31.5912424
H	44.8018050	47.0635754	32.0588304
N	46.2787199	48.0841382	33.2019562
H	47.1700804	47.8993699	33.7569761
C	45.4889529	49.0391755	33.7129130
N	44.2185583	49.2263786	33.2786032
H	43.9676321	48.9499603	32.3365918
H	43.7453147	50.0474948	33.6509883
N	45.9554891	49.8016654	34.6933138
H	46.9193651	49.6477444	35.0859102
H	45.4085341	50.6106582	34.9792560
S	49.0985513	48.4931234	44.4975383
N	47.7852807	47.5309382	42.2084174
C	48.1168471	46.2035837	42.1621375
C	46.4323917	47.5748274	42.4478380
C	46.9402513	45.3781721	42.3954554
C	45.8742622	46.2269641	42.5814144
C	45.7123366	48.7664605	42.5442926
H	44.6311735	48.7050437	42.6556022
C	46.2243426	50.0635349	42.5488267
N	47.5582514	50.3926743	42.5305695
C	47.6209888	51.7582667	42.6842829
C	46.2845459	52.3137946	42.7539797
C	45.4096255	51.2582597	42.6775913
C	48.8084544	52.4848060	42.7724442
H	48.7319196	53.5449308	42.9967067
C	50.0877039	51.9683845	42.6005577
C	51.3080194	52.7527524	42.5995118
C	51.7343443	50.5712441	42.2479258
N	50.3706274	50.6440223	42.3659697
C	52.3397346	51.8839076	42.3965428
C	52.4565149	49.4153651	41.9761503
H	53.5390792	49.5294860	41.9317423
C	51.9403401	48.1405548	41.7517199
C	52.7246312	46.9799881	41.3998414
N	50.6052191	47.8096939	41.8058061
C	50.5440027	46.4337344	41.6113528
C	49.3904050	45.7032071	41.8830501
H	49.5048244	44.6287845	41.9562262
C	51.8591441	45.9105767	41.2607740
Fe	49.0774606	49.0951030	42.3274936
O	48.7998312	49.5052153	40.2412461
H	49.9917879	48.0835878	40.0440343
O	49.4175959	48.5321334	39.3822132
O	48.4493681	49.3637260	35.6746192
C	48.8954536	48.1968846	35.4696946
O	48.3893078	47.3854523	34.6573232
C	50.1376580	47.7457102	36.2687113
C	51.1424397	48.8782620	36.5643496
C	52.3717019	48.4881185	37.4287711

C	53.4311130	49.6094633	37.5844805
O	47.4871227	51.3572319	37.2572497
H	47.9411022	50.6344829	36.7709689
H	46.9752233	50.8569793	37.9562877
O	49.6501242	51.5410127	38.9602016
H	49.1970647	50.3849087	39.8774939
H	48.8994149	51.5879001	38.3198879
H	46.0148599	57.0604097	40.1853782
H	45.8325874	46.0291166	33.0572224
H	49.6628020	49.5036534	45.2490685
H	46.9313884	44.2972736	42.2552880
H	44.8285598	46.0325845	42.8197216
H	46.0364419	53.3676648	42.8799530
H	44.3221223	51.2921592	42.6123633
H	51.3895305	53.8357824	42.6915401
H	53.4006980	52.1144450	42.3002291
H	53.8105659	46.9975819	41.3076275
H	52.1795441	44.9346629	40.8960778
H	49.7643797	47.3354544	37.2070186
H	50.6113974	46.9173488	35.7419708
H	51.4875345	49.2974860	35.6192423
H	50.6090426	49.6854269	37.0663851
H	52.0236566	48.1802587	38.4147497
H	52.8522703	47.6043875	37.0090800
H	54.2674460	49.2634571	38.1918469
H	53.8454963	49.8748283	36.6118886
H	53.0470829	50.5217907	38.0408210

$TS_{O\text{-}O}$

C	46.3778367	56.2122442	39.6522431
H	45.7553504	55.3087922	39.6939510
H	46.2197743	56.6283745	38.6367318
C	47.7708543	55.6854386	39.7920137
N	48.9436252	56.3874442	39.9761004
H	49.0645121	57.3517263	40.2912300
C	49.9748564	55.5037100	39.8834600
H	51.0077056	55.8094006	40.0434228
N	49.5428402	54.2831337	39.6363048
H	49.9051150	52.4674410	39.4537241
C	48.1755611	54.3799915	39.5818354
H	47.5535510	53.5107079	39.3692171
C	45.8800200	46.9671105	32.4513853
H	46.5590787	46.7777994	31.6032959
H	44.8685213	47.0952658	32.0436864
N	46.3404788	48.1190041	33.1999442
H	47.2695363	47.9517921	33.7046923
C	45.5540949	49.0585941	33.7402468
N	44.2693765	49.2300228	33.3354065
H	44.0137774	48.9707200	32.3896469
H	43.7967518	50.0471205	33.7186056
N	46.0335109	49.8190478	34.7170301
H	47.0112514	49.6758811	35.0850130
H	45.4872482	50.6223087	35.0172553
S	49.0181529	48.5694115	44.4098995
N	47.6933984	47.6165650	42.1623733
C	48.0294379	46.2859179	42.1320674
C	46.3365254	47.6636830	42.4133999
C	46.8587011	45.4684914	42.3839674
C	45.7898509	46.3197753	42.5677841
C	45.6082393	48.8476685	42.5073838
H	44.5267010	48.7821590	42.6062669
C	46.1210259	50.1405479	42.5216920
N	47.4515725	50.4634338	42.5271867

C	47.5263747	51.8259493	42.6591543
C	46.1907828	52.3898199	42.7244815
C	45.3109423	51.3401099	42.6444808
C	48.7153160	52.5470457	42.7258439
H	48.6451346	53.6105632	42.9304049
C	49.9876620	52.0247269	42.5386404
C	51.2063417	52.8044711	42.5349119
C	51.6414265	50.6292753	42.1569694
N	50.2780084	50.6989666	42.2961541
C	52.2401835	51.9370146	42.3179084
C	52.3639487	49.4828660	41.8623232
H	53.4437325	49.5994219	41.7890900
C	51.8372145	48.2161390	41.6451417
C	52.6115456	47.0554822	41.2660324
N	50.5129137	47.8833121	41.7550497
C	50.4384503	46.5163321	41.5492123
C	49.2975474	45.7819874	41.8496107
H	49.4184543	44.7097345	41.9405566
C	51.7433272	45.9890202	41.1557672
Fe	48.9821588	49.1732891	42.1568616
O	48.6799779	49.5877544	40.4228575
H	49.8138184	47.9116973	39.7257891
O	49.4272637	48.4617640	39.0220267
O	48.5496650	49.3880652	35.5988958
C	49.0279850	48.2469462	35.3414428
O	48.5428166	47.4613634	34.4890339
C	50.2778022	47.7872885	36.1200528
C	51.2130713	48.9326594	36.5542915
C	52.3711266	48.5377003	37.5105399
C	53.4321410	49.6484815	37.7190250
O	47.7441278	51.3032027	37.3390881
H	48.1660708	50.5725320	36.8359047
H	47.1728464	50.8133224	37.9961066
O	49.7925073	51.5324942	39.1623155
H	49.2009890	50.3810962	40.0378810
H	49.0972230	51.5609578	38.4615869
H	45.9421750	56.9049139	40.3722950
H	45.8839908	46.0609216	33.0570857
H	49.6208422	49.5582199	45.1604798
H	46.8485403	44.3856516	42.2597826
H	44.7474468	46.1223257	42.8177331
H	45.9469739	53.4414318	42.8752691
H	44.2234296	51.3749130	42.5798892
H	51.2943090	53.8850895	42.6471299
H	53.3015506	52.1668330	42.2243647
H	53.6962238	47.0681442	41.1593553
H	52.0515799	45.0108843	40.7865821
H	49.9022314	47.2630575	36.9987995
H	50.8062325	47.0407196	35.5272093
H	51.6208658	49.4048629	35.6605384
H	50.6141983	49.6960008	37.0510090
H	51.9372258	48.2606283	38.4712836
H	52.8690277	47.6399695	37.1441506
H	54.2040851	49.3162553	38.4131397
H	53.9358325	49.8680180	36.7776612
H	53.0261611	50.5842444	38.1032063

IM

C	46.4324732	56.3716576	39.4799995
H	45.7807202	55.4962933	39.3727723
H	46.3576485	56.9046453	38.5100786
C	47.7983166	55.7866991	39.6543687
N	48.9704858	56.4305243	39.9879082

H	49.1011147	57.3569763	40.4069727
C	49.9791018	55.5215653	39.8998210
H	51.0048594	55.7797865	40.1599978
N	49.5390292	54.3404401	39.5144682
H	49.8914763	52.4326962	39.3478166
C	48.1839342	54.4910729	39.3635297
H	47.5565691	53.6676503	39.0217399
C	45.8302523	46.9669883	32.4055051
H	46.4909320	46.7642908	31.5467340
H	44.8051784	47.0542774	32.0232930
N	46.2757893	48.1625698	33.0893940
H	47.2714155	48.0920722	33.4705317
C	45.4827138	49.0754970	33.6622398
N	44.1773404	49.2024479	33.3108966
H	43.8957574	48.9482303	32.3707744
H	43.6874697	49.9910041	33.7296904
N	45.9747202	49.8530673	34.6176709
H	46.9526730	49.7073506	34.9816756
H	45.4222973	50.6520265	34.9200794
S	49.0005697	48.5189236	44.5117183
N	47.7053966	47.5540209	42.2430192
C	48.0404414	46.2240439	42.2079752
C	46.3464793	47.6023411	42.4730017
C	46.8641205	45.4075994	42.4354531
C	45.7946275	46.2597789	42.6115244
C	45.6203249	48.7897237	42.5593027
H	44.5380613	48.7285057	42.6528503
C	46.1384955	50.0796363	42.5727325
N	47.4722767	50.3995144	42.5808656
C	47.5494875	51.7609985	42.7209315
C	46.2160991	52.3278211	42.7799313
C	45.3334170	51.2814151	42.6953237
C	48.7395452	52.4791521	42.8045050
H	48.6724374	53.5394306	43.0275965
C	50.0111951	51.9560792	42.6144607
C	51.2291269	52.7352342	42.6086115
C	51.6683052	50.5585899	42.2460069
N	50.3005132	50.6297830	42.3723292
C	52.2635391	51.8677799	42.3959314
C	52.3952358	49.4087556	41.9822046
H	53.4760624	49.5245556	41.9206262
C	51.8688160	48.1383513	41.7786917
C	52.6427110	46.9748167	41.4083046
N	50.5463018	47.8097704	41.8856950
C	50.4640956	46.4462068	41.6690714
C	49.3119495	45.7175154	41.9424619
H	49.4227562	44.6434130	42.0188443
C	51.7704034	45.9136776	41.2849801
Fe	49.0017060	49.1172645	42.2121529
O	48.7381778	49.4346962	40.4499807
H	49.9676766	47.7145992	39.7418549
O	49.8419471	48.2709994	38.9531622
O	48.4805543	49.3758504	35.5108928
C	49.0670068	48.3516804	35.0609235
O	48.6946199	47.7212684	34.0375802
C	50.3166751	47.8516319	35.8147180
C	51.2812195	48.9812273	36.2366363
C	52.5067439	48.5452991	37.0853102
C	53.5325181	49.6731148	37.3610585
O	47.6425015	51.3081615	37.2694853
H	48.0743285	50.5839521	36.7686753
H	47.1250793	50.8131641	37.9665999

O	49.7844616	51.4938263	39.0794130
H	49.2421090	50.2167876	40.0684550
H	49.0574258	51.5115146	38.4165501
H	45.9916950	56.9997040	40.2541697
H	45.8802922	46.0861007	33.0455053
H	49.6058370	49.5086977	45.2589440
H	46.8525978	44.3266713	42.2957071
H	44.7486135	46.0630903	42.8465467
H	45.9765196	53.3832550	42.9092684
H	44.2466647	51.3229030	42.6224169
H	51.3156901	53.8178275	42.7011625
H	53.3220419	52.1022187	42.2833375
H	53.7260900	46.9913382	41.2896279
H	52.0773372	44.9385447	40.9068601
H	49.9519802	47.3452327	36.7083759
H	50.8235267	47.1006841	35.2087098
H	51.6284243	49.4991183	35.3425978
H	50.7141570	49.7160257	36.8081068
H	52.1391031	48.1507932	38.0325549
H	53.0182561	47.7162337	36.5963592
H	54.3658568	49.2958793	37.9537701
H	53.9603863	50.0298372	36.4241780
H	53.1198287	50.5361318	37.8835107

TS$_{Hp\text{-}Od\text{-}recomb}$

C	46.3856538	56.2845119	39.6106139
H	45.8095263	55.3517533	39.6819968
H	46.1576101	56.6887261	38.6034728
C	47.8156027	55.8452610	39.6891816
N	48.9271112	56.5674182	40.0634967
H	48.9617443	57.4733306	40.5427443
C	50.0166247	55.7712854	39.8776825
H	51.0186575	56.0969993	40.1559933
N	49.6830464	54.5931319	39.3890789
H	50.0451946	52.6312795	38.9604657
C	48.3167195	54.6245802	39.2743779
H	47.7573691	53.7796672	38.8708694
C	45.8425058	46.9903321	32.4151722
H	46.4974581	46.7900903	31.5513078
H	44.8156179	47.0865861	32.0392556
N	46.2988151	48.1762511	33.1092981
H	47.2852813	48.0863836	33.5111405
C	45.5108286	49.0913696	33.6884782
N	44.2090203	49.2348611	33.3285767
H	43.9376902	49.0007204	32.3801787
H	43.7301006	50.0300361	33.7490711
N	46.0059061	49.8511566	34.6552611
H	46.9782648	49.6897151	35.0293667
H	45.4650369	50.6485937	34.9804507
S	48.9659139	48.4839375	44.5618061
N	47.6276288	47.4920456	42.3470663
C	47.9586663	46.1662376	42.2676192
C	46.2713103	47.5405628	42.5817341
C	46.7800532	45.3464205	42.4733197
C	45.7155335	46.1955053	42.6832949
C	45.5483683	48.7280224	42.6852273
H	44.4679438	48.6673210	42.8014105
C	46.0647730	50.0193308	42.6690344
N	47.3988696	50.3400700	42.6290317
C	47.4770558	51.7067828	42.7404025
C	46.1461166	52.2726373	42.8296766
C	45.2627970	51.2228278	42.7920943

C	48.6657452	52.4294835	42.7830125
H	48.5980726	53.4956437	42.9749175
C	49.9375177	51.9063914	42.5952778
C	51.1528983	52.6922400	42.5677275
C	51.5941853	50.5079428	42.2582548
N	50.2275710	50.5754182	42.3909734
C	52.1878113	51.8227623	42.3723732
C	52.3204458	49.3555553	42.0108628
H	53.4014974	49.4680247	41.9471336
C	51.7909892	48.0839331	41.8184710
C	52.5603117	46.9193430	41.4504277
N	50.4628928	47.7619189	41.9089872
C	50.3797251	46.3911904	41.7046340
C	49.2294399	45.6643466	41.9844038
H	49.3379180	44.5890787	42.0498816
C	51.6856244	45.8585076	41.3268575
Fe	48.9225601	49.0528883	42.2723910
O	48.5858374	49.3504942	40.5251862
H	50.2488372	47.7241071	39.7122847
O	50.0048429	48.3819033	39.0357295
O	48.4943606	49.3611897	35.5879004
C	49.0753348	48.3401920	35.1280968
O	48.6884593	47.7012593	34.1142184
C	50.3451871	47.8507976	35.8570701
C	51.3370055	48.9847191	36.1995094
C	52.5847105	48.5623978	37.0233430
C	53.6228637	49.6881388	37.2628358
O	47.4163504	51.3704336	37.1997863
H	47.9472549	50.6609922	36.7854070
H	46.9732724	50.8944542	37.9578776
O	49.9606751	51.7225727	38.6138875
H	49.4041561	49.3667028	39.9033457
H	49.0571130	51.6819782	38.2494012
H	45.9443142	56.9605409	40.3429194
H	45.8889543	46.1036349	33.0473729
H	49.5755248	49.4878029	45.2863585
H	46.7656954	44.2732196	42.2833172
H	44.6693152	45.9945790	42.9137793
H	45.9057764	53.3291134	42.9486810
H	44.1746520	51.2641964	42.7441745
H	51.2396948	53.7754411	42.6526335
H	53.2445305	52.0568870	42.2435424
H	53.6421406	46.9350301	41.3182638
H	51.9951236	44.8827281	40.9525158
H	50.0063520	47.3881678	36.7840176
H	50.8219556	47.0692928	35.2654543
H	51.6643884	49.4585233	35.2740921
H	50.8006854	49.7550574	36.7535912
H	52.2463353	48.1782010	37.9856123
H	53.0808952	47.7224546	36.5371709
H	54.4510247	49.3209428	37.8689576
H	54.0552299	50.0140708	36.3168469
H	53.2127004	50.5698530	37.7551905

Cpd I

C	46.3622170	56.2791303	39.6385530
H	45.8359031	55.3261072	39.7982356
H	46.0468125	56.6162791	38.6314319
C	47.8197408	55.9227385	39.6335533
N	48.8934353	56.6294244	40.1254131
H	48.8865401	57.5078414	40.6540572
C	50.0195294	55.9110932	39.8587252
H	51.0037316	56.2457083	40.1859453

N	49.7420371	54.7933717	39.2170690
H	50.1208109	52.9168251	38.5332697
C	48.3780927	54.7869224	39.0747566
H	47.8608253	53.9726423	38.5654604
C	45.8481797	46.9925596	32.4085433
H	46.5031037	46.7925249	31.5446815
H	44.8193608	47.0747449	32.0343631
N	46.2940365	48.1885535	33.0905354
H	47.2990110	48.1312212	33.4580546
C	45.5016814	49.0948608	33.6757067
N	44.1949222	49.2251049	33.3246511
H	43.9267925	49.0025220	32.3722198
H	43.7125020	50.0153399	33.7503868
N	45.9960940	49.8555851	34.6415936
H	46.9732045	49.6966893	35.0080529
H	45.4601707	50.6574807	34.9639046
S	48.8847457	48.4518651	44.6799142
N	47.5510441	47.4614265	42.3952460
C	47.8765666	46.1396670	42.3472648
C	46.1949094	47.5079888	42.6062415
C	46.6969502	45.3137658	42.5460797
C	45.6293691	46.1644730	42.7191458
C	45.4843408	48.7070568	42.6957720
H	44.4019254	48.6504075	42.8014939
C	46.0004447	50.0056589	42.6928992
N	47.3340153	50.3135040	42.6636686
C	47.4089033	51.6785579	42.7792441
C	46.0810572	52.2535533	42.8648521
C	45.1951344	51.2068001	42.8187919
C	48.6049381	52.3974868	42.8077820
H	48.5381865	53.4653445	42.9985103
C	49.8858763	51.8833867	42.6203649
C	51.0984601	52.6782724	42.5715844
C	51.5268362	50.4925427	42.2803125
N	50.1657353	50.5560096	42.4296308
C	52.1300670	51.8087952	42.3686251
C	52.2419926	49.3257426	42.0506188
H	53.3222479	49.4402882	41.9598919
C	51.7230855	48.0375302	41.8984593
C	52.5019007	46.8740548	41.5367450
N	50.4086324	47.7143925	42.0315421
C	50.3264512	46.3570982	41.8190809
C	49.1633186	45.6414765	42.1038778
H	49.2684729	44.5660756	42.1835492
C	51.6291148	45.8120900	41.4317755
Fe	48.8440298	49.0250257	42.2549947
O	48.6221488	49.1841596	40.6319558
H	50.4854765	47.0532103	39.5040334
O	50.3510362	47.8412573	38.9625377
O	48.4980200	49.3986519	35.5421417
C	49.1230202	48.4368872	35.0160752
O	48.7388363	47.8220699	33.9838388
C	50.4354545	47.9837197	35.6851348
C	51.4147105	49.1354596	36.0121810
C	52.6800274	48.7157345	36.8128539
C	53.7165144	49.8399562	37.0599550
O	47.3396346	51.4261232	37.1186367
H	47.9176791	50.7647948	36.6886197
H	46.9762291	50.9176329	37.8962258
O	50.0414648	52.0982259	38.0101560
H	49.7538703	48.4038930	39.5061370
H	49.0861485	51.9221353	37.9515251

H	45.9234106	56.9671619	40.3611377
H	45.9070232	46.1112302	33.0471848
H	49.5162798	49.4422070	45.4043001
H	46.6900642	44.2368899	42.3776814
H	44.5784895	45.9695737	42.9330001
H	45.8469314	53.3117244	42.9811545
H	44.1072222	51.2498484	42.7672059
H	51.1817518	53.7620937	42.6519650
H	53.1855876	52.0365172	42.2200109
H	53.5806342	46.8992808	41.3825865
H	51.9309402	44.8340496	41.0570684
H	50.1529180	47.5000311	36.6201672
H	50.9129882	47.2273039	35.0623476
H	51.7182794	49.6198280	35.0841183
H	50.8788058	49.8983909	36.5768128
H	52.3440154	48.3168408	37.7699573
H	53.1747526	47.8871658	36.3061114
H	54.5413116	49.4686312	37.6681445
H	54.1546602	50.1767584	36.1204553
H	53.3046941	50.7209568	37.5522039

Cpd 0 to Cpd I formation via Arg245

Cpd 0

C	45.9906799	47.0102599	32.5126266
H	46.6315344	46.8175798	31.6367491
H	44.9835498	47.2439339	32.1448123
N	46.5566872	48.0874120	33.3070000
H	47.4396785	47.8244303	33.7861946
C	45.8361085	49.0064072	33.9972362
N	44.5778368	49.3122294	33.6097558
H	44.3360550	49.1781729	32.6349416
H	44.1614163	50.1353375	34.0532375
N	46.3766083	49.5656880	35.0588599
H	47.3225932	49.2566359	35.3339420
H	46.0288828	50.3592150	35.6508612
S	49.0679324	48.5001325	44.4186771
N	47.5780284	47.6761286	42.1320218
C	47.9165395	46.3545996	42.0678956
C	46.2367726	47.7120988	42.4184586
C	46.7522228	45.5189186	42.3305339
C	45.6877472	46.3596894	42.5533087
C	45.5204778	48.8993553	42.5770893
H	44.4403886	48.8356893	42.7007506
C	46.0415527	50.1911053	42.6366410
N	47.3736185	50.5050703	42.6484914
C	47.4580614	51.8569349	42.8444357
C	46.1267102	52.4302973	42.9055885
C	45.2389058	51.3907881	42.7906322
C	48.6629004	52.5554553	42.9715940
H	48.6016679	53.5981017	43.2685118
C	49.9363991	52.0478797	42.7062998
C	51.1580372	52.8348451	42.6497904
C	51.5541390	50.6792160	42.1523033
N	50.2050281	50.7489251	42.3699236
C	52.1751827	51.9786098	42.3327871
C	52.2566806	49.5400854	41.7732104
H	53.3364123	49.6529677	41.6845622
C	51.7252005	48.2795057	41.5177918
C	52.5060863	47.1094357	41.1702092
N	50.3927252	47.9624254	41.5910753
C	50.3234606	46.5913699	41.4226197
C	49.1871014	45.8604147	41.7588558
H	49.3142123	44.7891189	41.8657902

C	51.6356202	46.0457664	41.0682774
Fe	48.8697818	49.2450611	42.2037658
O	48.4510477	49.9168687	40.3999776
H	49.7218255	48.6690036	39.7262540
O	48.9713314	49.1230097	39.2946070
O	45.9477027	50.2606010	39.3998138
H	45.3795684	49.4631589	39.3803513
H	46.7996149	49.9830054	39.8325950
O	46.2827866	51.4311088	36.9996220
H	47.2746965	51.4023550	37.0555201
H	46.0603950	50.9744813	37.8568271
O	48.9652783	51.0351894	37.3013964
H	49.0412487	50.4592880	38.0994954
H	49.0101945	50.3118958	36.5899775
H	45.9413833	46.0783011	33.0757339
H	49.6313248	49.4795213	45.2109739
H	46.7457424	44.4389370	42.1833439
H	44.6503908	46.1577569	42.8201082
H	45.8942160	53.4857517	43.0471143
H	44.1532494	51.4341978	42.7038394
H	51.2405726	53.9178296	42.7414408
H	53.2283851	52.2140394	42.1798013
H	53.5930123	47.1073291	41.0886332
H	51.9503420	45.0577800	40.7322712

TS1

C	46.0501494	47.0983563	32.5355424
H	46.6804492	46.9029333	31.6491484
H	45.0422631	47.3451316	32.1781977
N	46.6242017	48.1730972	33.3246958
H	47.4938429	47.8893660	33.8091945
C	45.8721585	49.0358029	34.1053236
N	44.5965038	49.3169201	33.6539296
H	44.5226163	49.4080005	32.6440848
H	44.2017146	50.1307719	34.1321516
N	46.3222702	49.5470531	35.2053658
H	47.2653336	49.1779859	35.4135096
H	46.2199195	50.6138207	36.4016582
S	49.0397707	48.5133302	44.4055339
N	47.5447149	47.6710364	42.1453279
C	47.8802496	46.3484991	42.0784063
C	46.2023345	47.7065809	42.4349621
C	46.7156588	45.5134519	42.3385104
C	45.6533059	46.3540946	42.5683708
C	45.4871257	48.8938108	42.5892719
H	44.4081719	48.8312583	42.7224031
C	46.0095523	50.1866794	42.6359667
N	47.3439407	50.5017026	42.6345832
C	47.4278623	51.8575666	42.8232319
C	46.0983865	52.4280344	42.8885780
C	45.2093927	51.3861475	42.7866363
C	48.6313475	52.5596895	42.9419907
H	48.5705291	53.6023678	43.2398047
C	49.9027590	52.0515948	42.6763715
C	51.1269063	52.8345220	42.6316823
C	51.5186662	50.6788407	42.1313222
N	50.1672363	50.7497745	42.3380492
C	52.1427027	51.9756560	42.3183412
C	52.2208909	49.5367569	41.7625873
H	53.3008448	49.6472904	41.6767016
C	51.6874726	48.2765644	41.5165023
C	52.4666653	47.1050088	41.1780645
N	50.3507401	47.9606208	41.5890878

C	50.2851261	46.5841301	41.4325610
C	49.1506060	45.8535923	41.7712342
H	49.2772774	44.7827488	41.8805362
C	51.5966008	46.0400856	41.0817821
Fe	48.8406326	49.2321393	42.2340932
O	48.3583463	49.8927241	40.3302117
H	49.6926360	48.6565448	39.7750023
O	49.0226782	49.1577797	39.2637274
O	46.1149520	50.0985243	39.3650842
H	45.4861278	49.3411850	39.3337708
H	47.0684567	49.8708433	39.8530487
O	46.4398716	51.1967761	37.2295861
H	47.4466121	51.2425530	37.2174810
H	46.2346063	50.5688374	38.3168212
O	49.0614092	51.0366865	37.3075618
H	49.1995828	50.4695066	38.1020667
H	49.0575989	50.2836524	36.5990244
H	45.9910494	46.1523172	33.0736729
H	49.6099150	49.4985519	45.1856755
H	46.7083215	44.4348084	42.1818530
H	44.6171918	46.1517074	42.8396163
H	45.8660113	53.4834107	43.0308795
H	44.1229288	51.4299271	42.7108290
H	51.2141465	53.9163016	42.7327224
H	53.1977501	52.2075698	42.1728822
H	53.5535412	47.1010725	41.0958908
H	51.9130558	45.0510866	40.7504148

RC

C	46.0554151	47.0842132	32.5164080
H	46.6816106	46.8712872	31.6306321
H	45.0372347	47.2881834	32.1605924
N	46.6015202	48.2075197	33.2527888
H	47.4978869	47.9888851	33.7207491
C	45.8292839	49.0730492	34.0158020
N	44.5406797	49.2990125	33.5549299
H	44.4799835	49.4020692	32.5443123
H	44.1272880	50.1015876	34.0367688
N	46.2563804	49.6397465	35.0928603
H	47.1951981	49.2937962	35.3509634
H	46.1448902	50.8162479	36.3357955
S	49.0595668	48.4391380	44.5632843
N	47.6386529	47.5230821	42.2978189
C	47.9682181	46.1994271	42.2049792
C	46.2901775	47.5588921	42.5646443
C	46.7952132	45.3657526	42.4286449
C	45.7331226	46.2068911	42.6596359
C	45.5735162	48.7462988	42.7077733
H	44.4946670	48.6847604	42.8398732
C	46.0906723	50.0407918	42.7176777
N	47.4258988	50.3607686	42.6913034
C	47.4994172	51.7244944	42.8427255
C	46.1690057	52.2889041	42.9106755
C	45.2854688	51.2399124	42.8446331
C	48.6941583	52.4406640	42.9360973
H	48.6263207	53.4894774	43.2137690
C	49.9695953	51.9319924	42.7009322
C	51.1827117	52.7271738	42.6291047
C	51.6020760	50.5532306	42.2382316
N	50.2475104	50.6160931	42.4325752
C	52.2092315	51.8662019	42.3646064
C	52.3131866	49.4043891	41.9119410
H	53.3922339	49.5200366	41.8197745

C	51.7835805	48.1381847	41.6860354
C	52.5553410	46.9751398	41.3152421
N	50.4446241	47.8163037	41.7685071
C	50.3782828	46.4399832	41.5825735
C	49.2377326	45.7068129	41.8935367
H	49.3560576	44.6327495	41.9652312
C	51.6846330	45.9098070	41.2047895
Fe	48.9373172	49.0754675	42.4106817
O	48.5429804	49.6482342	40.3749390
H	49.8792045	48.3892305	39.9847969
O	49.2914033	48.9061202	39.3879957
O	46.2085237	49.9132452	39.3951711
H	45.5017364	49.2350098	39.3034324
H	47.5789759	49.6070103	40.0075130
O	46.3687453	51.3901979	37.1355266
H	47.3412755	51.2875600	37.2047370
H	46.1920427	50.4129135	38.5336555
O	49.1187527	51.0290827	37.6196926
H	49.3883584	50.4244145	38.3400400
H	49.0110590	50.3186769	36.8756722
H	46.0254938	46.1575510	33.0895303
H	49.6218189	49.4519110	45.3132914
H	46.7836920	44.2912852	42.2457402
H	44.6898195	46.0045259	42.9017808
H	45.9341708	53.3478145	43.0185104
H	44.1988872	51.2814319	42.7692432
H	51.2580160	53.8115659	42.7096666
H	53.2626671	52.1039726	42.2169395
H	53.6389427	46.9807889	41.1975867
H	51.9998486	44.9316329	40.8416342

TS$_{O-O}$

C	46.1072152	47.1141586	32.4846151
H	46.7376627	46.8777209	31.6084054
H	45.0868340	47.2895653	32.1192059
N	46.6420041	48.2759903	33.1665156
H	47.5899877	48.1202576	33.5499233
C	45.8758633	49.1066967	33.9756409
N	44.5552002	49.2754799	33.5745332
H	44.4534276	49.3807408	32.5671217
H	44.1362721	50.0664109	34.0708646
N	46.3241993	49.6934714	35.0306891
H	47.2918542	49.3985565	35.2439043
H	46.1185363	50.8469787	36.3541166
S	48.9415459	48.4541700	44.6528127
N	47.6230474	47.5952974	42.2720732
C	47.9626220	46.2772549	42.2039525
C	46.2691346	47.6127929	42.5118319
C	46.7978194	45.4281110	42.4158795
C	45.7219976	46.2595747	42.6146815
C	45.5488996	48.7998158	42.6411374
H	44.4638032	48.7387003	42.7180741
C	46.0735255	50.0946490	42.7110932
N	47.4053834	50.3796994	42.7798840
C	47.4968145	51.7393327	42.9455173
C	46.1726569	52.3308391	42.9543736
C	45.2774113	51.3017198	42.8157232
C	48.7017721	52.4335693	43.0662561
H	48.6298825	53.4846856	43.3332669
C	49.9927120	51.9464002	42.8336464
C	51.1919319	52.7620042	42.7487761
C	51.6386821	50.5989938	42.3721819
N	50.2889221	50.6381582	42.5762768

C	52.2320574	51.9161600	42.4849872
C	52.3501497	49.4501645	42.0318713
H	53.4280926	49.5708669	41.9231987
C	51.8233052	48.1829347	41.7941817
C	52.5896493	47.0275508	41.3781577
N	50.4948097	47.8707456	41.9060301
C	50.4090249	46.5270005	41.6482739
C	49.2514521	45.7978352	41.9401812
H	49.3750560	44.7238566	42.0254478
C	51.7071715	45.9782965	41.2497899
Fe	48.9415521	49.1460000	42.3533775
O	48.6955365	49.8189852	40.6321587
H	50.3847083	48.7655966	39.6277816
O	49.7851466	48.4660557	38.9194448
O	46.0975999	50.0185573	39.5165908
H	45.4575443	49.2891678	39.3714029
H	47.7903504	49.6335820	40.2902485
O	46.3216862	51.4102736	37.1568813
H	47.2866040	51.2424696	37.2799426
H	46.1006225	50.4759374	38.6385701
O	48.9645732	50.8479525	37.6157144
H	49.1810808	50.1622806	38.2873447
H	48.9824027	50.2524161	36.7824417
H	46.0874270	46.2037164	33.0835897
H	49.5552371	49.4082330	45.4386206
H	46.8042235	44.3513916	42.2464646
H	44.6776146	46.0506245	42.8463517
H	45.9453189	53.3907272	43.0684861
H	44.1979130	51.3581641	42.6758497
H	51.2528944	53.8485373	42.8103911
H	53.2819188	52.1624005	42.3261671
H	53.6719870	47.0332211	41.2493939
H	52.0028036	45.0020213	40.8656604

Cpd 0 to Cpd I formation via Substrate

Cpd 0

C	46.7140131	56.6600159	39.0800110
H	45.9900129	56.4980159	38.2820108
H	47.5220134	57.2540161	38.6530109
C	47.2500133	55.3190155	39.4120111
N	48.5080136	55.0250155	39.9640112
H	49.1240138	55.7280157	40.3490113
C	48.7440137	53.6970151	39.8510112
H	49.6000139	53.1440149	40.2080113
N	47.5870134	53.1250149	39.4660111
H	47.5160134	52.1980147	39.0710110
C	46.6740131	54.0850152	39.1290110
H	45.7050128	53.9530152	38.6700109
C	45.7140128	46.7560131	32.4650091
H	46.2220130	46.4200130	31.5610089
H	44.6750126	46.9740132	32.2170091
N	46.3050130	48.0200135	33.0270093
H	47.0720132	47.8200134	33.6530095
C	45.6100128	49.0880138	33.4550094
N	44.4840125	49.3930139	33.0340093
H	43.8530123	48.8120137	32.5000091
H	44.2000124	50.3370141	33.2550093
N	46.2200130	49.7900140	34.3770097
H	47.0140132	49.4160139	34.8760098
H	45.9570129	50.6560142	34.8260098
S	49.0110138	48.7400137	44.5910125
N	47.8710135	47.6730134	42.2610119
C	48.1310135	46.4450131	42.1940119

C	46.5770131	47.7690134	42.4530119
C	47.0410132	45.6850128	42.3460119
C	46.0870130	46.5280131	42.5570120
C	45.9420129	48.9140137	42.6320120
H	44.8860126	48.9500138	42.8900121
C	46.5590131	50.1450141	42.6460120
N	47.7930134	50.3980142	42.5510120
C	47.8710135	51.7010145	42.6840120
C	46.6830131	52.2990147	42.8470120
C	45.8540129	51.2640144	42.8070120
C	49.0270138	52.4510147	42.7680120
H	48.9590138	53.4970150	43.0610121
C	50.2020141	51.8990146	42.6250120
C	51.3130144	52.5740148	42.5040119
C	51.7320145	50.5280142	42.2950119
N	50.4560142	50.6330142	42.5170119
C	52.2970147	51.6930145	42.3260119
C	52.4530147	49.3710139	42.0090118
H	53.5320150	49.5000139	42.0480118
C	51.9100146	48.1250135	41.9230118
C	52.6400148	47.0720132	41.7090117
N	50.6320142	47.8860135	42.0080118
C	50.4930142	46.6450131	41.8050117
C	49.3510139	45.9060129	41.9970118
H	49.4810139	44.8280126	41.9370118
C	51.7370145	46.0650129	41.5900117
Fe	49.2220138	49.1180138	42.3400119
O	48.9200137	49.2900139	40.5250114
H	49.9400140	47.6810134	40.1040113
O	49.8050140	48.4780136	39.5610111
O	48.4420136	49.6030139	35.9980101
C	48.9320138	48.5480136	35.5950100
O	48.1480135	47.8310134	35.0160098
C	50.2430141	48.0010135	36.1710102
C	51.2880144	49.1460138	36.4560102
C	52.6750148	48.7440137	37.1360104
C	53.5250150	49.9850140	37.3080105
H	49.2300138	49.7000140	36.5760103
O	46.5070131	51.2260144	36.8420104
H	47.1860133	50.7330143	36.3800102
H	46.5390131	50.8940143	37.7400106
O	49.0870138	51.0980144	38.1710107
H	49.2750138	50.3110141	38.6830109
H	49.0450138	50.7900143	37.2660105
H	46.1814744	57.1793454	39.8767334
H	45.7601145	45.9212178	33.1643364
H	49.5132404	49.7504459	45.3854573
H	46.9599904	44.5983121	42.3218472
H	45.0297446	46.3707865	42.7703909
H	46.4178432	53.3497949	42.9636868
H	44.7698608	51.3766179	42.8083852
H	51.3785828	53.6615941	42.5346580
H	53.3427981	51.9790776	42.2138867
H	53.7108065	47.0670129	41.5054256
H	52.1167367	45.0651576	41.3798510
H	50.0352467	47.4619414	37.0952943
H	50.6734466	47.2828768	35.4731027
H	51.5104120	49.6379843	35.5091356
H	50.8059607	49.8865112	37.0942539
H	52.4963876	48.2744152	38.1032936
H	53.1977484	48.0133108	36.5188475
H	54.4570017	49.6848446	37.7869325

H	53.7811381	50.3512251	36.3138487
H	53.1156790	50.8213910	37.8745656

RC

C	46.5655993	56.6717437	39.4591998
H	45.9334254	56.6535454	38.5572086
H	47.4897580	57.2099573	39.1919114
C	46.9649284	55.2781325	39.8164151
N	48.2511071	54.9941387	40.2600258
H	48.9571607	55.7027386	40.5265245
C	48.4120549	53.6766501	40.3916764
H	49.3205981	53.1551999	40.6773557
N	47.2587607	53.0995732	40.0536089
H	47.0556402	52.0972693	40.0840604
C	46.3413248	54.0631541	39.6978592
H	45.3304128	53.7981628	39.4154049
C	45.8255882	46.9174038	32.4410845
H	46.5014253	46.7369280	31.5897698
H	44.8123416	47.0535011	32.0439682
N	46.2784245	48.0644094	33.2094139
H	47.1567030	47.8818579	33.7585087
C	45.4933918	49.0336995	33.6974561
N	44.2478149	49.2503299	33.2248018
H	43.9946152	48.9421496	32.2927873
H	43.7622951	50.0707813	33.5764757
N	45.9451116	49.7903548	34.6955979
H	46.8590567	49.5907769	35.1586786
H	45.3959498	50.6080305	34.9540138
S	49.1183726	48.3459816	44.7677246
N	47.8125196	47.3328223	42.4483743
C	48.1492313	46.0119978	42.3327724
C	46.4533195	47.3552141	42.6582535
C	46.9738401	45.1674066	42.5036421
C	45.8994426	45.9987470	42.7121168
C	45.7196437	48.5355662	42.7803318
H	44.6394002	48.4620351	42.8912303
C	46.2195695	49.8380008	42.8167025
N	47.5572210	50.1755531	42.7908135
C	47.6102371	51.5348941	43.0141390
C	46.2722358	52.0822167	43.0970747
C	45.4003125	51.0256552	42.9787106
C	48.7974019	52.2641979	43.1103960
H	48.7246307	53.3063177	43.4120159
C	50.0707480	51.7783587	42.8299252
C	51.2553392	52.6015502	42.6873474
C	51.7206948	50.4269670	42.3539020
N	50.3656529	50.4594346	42.5790929
C	52.2913318	51.7618206	42.3999204
C	52.4607984	49.2808712	42.0896967
H	53.5372522	49.4191197	41.9934599
C	51.9639055	47.9890858	41.9296417
C	52.7522816	46.8395257	41.5579096
N	50.6280625	47.6461052	42.0047222
C	50.5772114	46.2712376	41.7832865
C	49.4249178	45.5319182	42.0278265
H	49.5347530	44.4568555	42.0357339
C	51.8933052	45.7647227	41.4132858
Fe	49.0929552	48.8897174	42.6114983
O	48.7864314	49.2901050	40.4424928
H	50.1997158	48.0848378	40.3065610
O	49.7207167	48.5897667	39.5952328
O	48.3354029	49.1624696	35.9446540
C	48.8214934	48.0317585	35.6369599

O	48.4110406	47.3143305	34.7055211
C	50.0416868	47.5428730	36.4703166
C	51.1804399	48.5911747	36.5254243
C	52.4129630	48.2484966	37.4094794
C	53.4827221	49.3725728	37.4839664
H	50.4203305	50.7223075	39.0588189
O	47.6269235	50.9636369	37.7397961
H	47.9043333	50.1895629	37.1910700
H	47.0318912	50.5937811	38.4402213
O	49.7935689	51.4470795	39.2018886
H	49.0823970	50.2239157	40.2813369
H	49.0475329	51.2851190	38.5556637
H	46.0296357	57.2127267	40.2390380
H	45.8320446	46.0143926	33.0514912
H	49.6227748	49.3896475	45.5165151
H	46.9745867	44.0957744	42.3044662
H	44.8501816	45.7878385	42.9186022
H	46.0273411	53.1408053	43.1835853
H	44.3153003	51.0684385	42.8839199
H	51.2976152	53.6896275	42.7360049
H	53.3361889	52.0174177	42.2238214
H	53.8367920	46.8649624	41.4518083
H	52.2268780	44.7998904	41.0313238
H	49.6958392	47.3378726	37.4834461
H	50.3953469	46.5939371	36.0671883
H	51.5158522	48.8031852	35.5102311
H	50.7382916	49.5254879	36.8713012
H	52.0766988	48.0057031	38.4174699
H	52.8769461	47.3317048	37.0457768
H	54.3421247	49.0416824	38.0670666
H	53.8608050	49.6100750	36.4896259
H	53.1167877	50.2973113	37.9300808

TS1

C	46.5685024	56.6059441	39.5005579
H	45.9466255	56.6029773	38.5909298
H	47.4995546	57.1381125	39.2456428
C	46.9602794	55.2016246	39.8332722
N	48.2665968	54.8948826	40.1969957
H	48.9856288	55.5951258	40.4498243
C	48.4243393	53.5719239	40.2793384
H	49.3468976	53.0325887	40.4806406
N	47.2482704	53.0132974	39.9936722
H	47.0464970	52.0066200	39.9803239
C	46.3210848	53.9931791	39.7185833
H	45.2938757	53.7441464	39.4832716
C	45.8094126	46.9202445	32.4249078
H	46.4798530	46.7374581	31.5696709
H	44.7935746	47.0524928	32.0330058
N	46.2648791	48.0711453	33.1845443
H	47.1413198	47.8870161	33.7385831
C	45.4809460	49.0387429	33.6790224
N	44.2271742	49.2482983	33.2241767
H	43.9600937	48.9312731	32.2991924
H	43.7465219	50.0712767	33.5766544
N	45.9440355	49.7979078	34.6687290
H	46.8859100	49.6268374	35.0838913
H	45.4042063	50.6195240	34.9340271
S	49.0584780	48.3417311	44.7911078
N	47.6825304	47.3525769	42.4891988
C	48.0105598	46.0272144	42.3552274
C	46.3284869	47.3764848	42.7196067
C	46.8315909	45.1894237	42.5216035

C	45.7662949	46.0238970	42.7591839
C	45.6043592	48.5626152	42.8664730
H	44.5252345	48.4929291	42.9912454
C	46.1063758	49.8622414	42.8855508
N	47.4446802	50.1883409	42.8608513
C	47.5045533	51.5561933	43.0483625
C	46.1681540	52.1095432	43.1164939
C	45.2927888	51.0561007	43.0229779
C	48.6927721	52.2804883	43.1322825
H	48.6225195	53.3281806	43.4138874
C	49.9695460	51.7930304	42.8524369
C	51.1544346	52.6098877	42.6968118
C	51.6042919	50.4342272	42.3691676
N	50.2544980	50.4734589	42.6093710
C	52.1850326	51.7620006	42.4043620
C	52.3298948	49.2789002	42.0971761
H	53.4049846	49.4127115	41.9798315
C	51.8269619	47.9880425	41.9415921
C	52.6133029	46.8430979	41.5439388
N	50.5013978	47.6422402	42.0534092
C	50.4435607	46.2855142	41.8053104
C	49.2824188	45.5463215	42.0516441
H	49.3895122	44.4701619	42.0631356
C	51.7514856	45.7715764	41.4087430
Fe	48.9639988	48.8952630	42.6191540
O	48.5981593	49.3686484	40.6854393
H	50.0345811	48.0779806	40.0857297
O	49.5704621	48.6233228	39.4183720
O	48.4127775	49.2670201	35.7950602
C	48.8525863	48.1036265	35.5519910
O	48.3795823	47.3295058	34.6969121
C	50.0830712	47.6299984	36.3758679
C	51.2138350	48.6842884	36.4471732
C	52.4207621	48.3465816	37.3675016
C	53.4956943	49.4634118	37.4562199
H	50.0237345	50.3715377	38.9969092
O	47.4784776	51.1562520	37.4206614
H	47.8656222	50.3958682	36.9260586
H	46.9641204	50.7340107	38.1522287
O	49.7847200	51.3212296	38.9661654
H	49.0117619	50.2447130	40.5341519
H	48.9964545	51.3308159	38.3717569
H	46.0296251	57.1544463	40.2731015
H	45.8224367	46.0187778	33.0374886
H	49.5666853	49.3835862	45.5398472
H	46.8242501	44.1220958	42.3006414
H	44.7181011	45.8154956	42.9734872
H	45.9286133	53.1699535	43.1953452
H	44.2073663	51.0981157	42.9326454
H	51.2044965	53.6978384	42.7406772
H	53.2278651	52.0099451	42.2066474
H	53.6954798	46.8677874	41.4160688
H	52.0773404	44.8080228	41.0170157
H	49.7307518	47.4179598	37.3853122
H	50.4432785	46.6844006	35.9706980
H	51.5744483	48.8880928	35.4389624
H	50.7664371	49.6214786	36.7781971
H	52.0477199	48.1268952	38.3678239
H	52.8872819	47.4197205	37.0338028
H	54.3385318	49.1331524	38.0633615
H	53.9005001	49.6868460	36.4691655
H	53.1212831	50.3938216	37.8830935

IM

C	46.5722514	56.5857229	39.5457465
H	45.9678205	56.5992343	38.6239839
H	47.5138739	57.1083214	39.3119437
C	46.9445564	55.1714998	39.8654091
N	48.2642085	54.8366632	40.1477849
H	49.0030734	55.5251818	40.3717090
C	48.4030795	53.5092120	40.2069372
H	49.3259428	52.9372853	40.3160377
N	47.2006942	52.9763685	39.9908504
H	46.9888529	51.9693586	39.9649613
C	46.2766410	53.9751701	39.7819198
H	45.2319838	53.7484009	39.6068444
C	45.8082208	46.9395743	32.4033447
H	46.4698903	46.7509610	31.5427455
H	44.7864863	47.0576788	32.0223733
N	46.2641109	48.1056876	33.1377275
H	47.1813555	47.9526428	33.6381233
C	45.4782346	49.0558240	33.6590830
N	44.2102524	49.2478492	33.2317531
H	43.9414930	48.9544606	32.2991902
H	43.7226197	50.0559598	33.6083632
N	45.9487862	49.8136588	34.6459639
H	46.8924877	49.6381908	35.0604356
H	45.4061403	50.6308085	34.9185615
S	49.0108962	48.3392762	44.8111640
N	47.6042897	47.3725106	42.5239265
C	47.9168535	46.0611914	42.3783569
C	46.2445409	47.4045820	42.7611091
C	46.7372119	45.2202504	42.5290159
C	45.6748285	46.0542201	42.7811811
C	45.5397873	48.5889079	42.9368066
H	44.4604710	48.5252163	43.0674446
C	46.0526889	49.8993192	42.9794735
N	47.3777622	50.2124376	42.9636970
C	47.4462229	51.5821847	43.1110782
C	46.1144130	52.1453493	43.1844295
C	45.2355769	51.0921065	43.1105460
C	48.6408855	52.2987909	43.1390173
H	48.5806822	53.3557460	43.3861808
C	49.9195694	51.8040843	42.8500452
C	51.1054229	52.6187588	42.6841485
C	51.5488490	50.4395713	42.3908538
N	50.1972056	50.4853483	42.6318830
C	52.1350353	51.7653070	42.4083153
C	52.2654643	49.2783270	42.1383422
H	53.3415843	49.4052178	42.0194497
C	51.7554229	47.9812288	41.9827276
C	52.5422428	46.8336073	41.5871984
N	50.4374305	47.6520356	42.0925485
C	50.3681026	46.2947024	41.8380442
C	49.2054959	45.5708947	42.0896514
H	49.3025439	44.4928430	42.1093616
C	51.6758804	45.7686106	41.4492178
Fe	48.8942184	48.9305682	42.5884394
O	48.5009900	49.3432908	40.7323768
H	50.1601106	48.1308822	40.0055304
O	49.9141398	48.6253249	39.2054141
O	48.4085099	49.2551404	35.7427513
C	48.9098574	48.1427280	35.4054190
O	48.4885222	47.4320343	34.4690402
C	50.1513893	47.6569180	36.1990932

C	51.2626154	48.7306606	36.2978040
C	52.4977774	48.3656436	37.1681971
C	53.5568253	49.4930351	37.2939286
H	50.1456494	50.3494159	39.0971346
O	47.5798055	51.1127946	37.4639985
H	47.9545538	50.3566295	36.9546739
H	47.0583655	50.6846534	38.1850334
O	49.9130907	51.3089503	39.0193652
H	48.9700764	50.1655807	40.5129637
H	49.1292497	51.2862123	38.4239584
H	46.0263134	57.1408301	40.3085537
H	45.8379046	46.0453552	33.0258864
H	49.5309481	49.3676321	45.5703593
H	46.7288976	44.1567274	42.2904435
H	44.6265971	45.8465199	42.9959805
H	45.8801831	53.2068883	43.2640447
H	44.1500633	51.1336037	43.0210739
H	51.1610175	53.7066602	42.7221862
H	53.1786009	52.0050180	42.2044120
H	53.6237094	46.8532057	41.4526142
H	51.9956644	44.8021026	41.0597727
H	49.8212065	47.4097636	37.2080338
H	50.5249389	46.7353490	35.7527320
H	51.5930393	48.9979633	35.2940927
H	50.8053679	49.6350281	36.6991908
H	52.1480683	48.0888618	38.1627636
H	52.9718879	47.4672671	36.7729680
H	54.4125865	49.1484964	37.8744741
H	53.9462093	49.7660567	36.3131603
H	53.1770316	50.4004896	37.7633432

TS2

C	46.5758376	56.5766916	39.5473491
H	45.9694871	56.5850222	38.6267258
H	47.5151038	57.1015971	39.3090531
C	46.9540390	55.1641772	39.8695958
N	48.2770287	54.8336484	40.1436160
H	49.0150418	55.5247522	40.3611457
C	48.4204703	53.5066403	40.2030818
H	49.3475129	52.9379770	40.2887583
N	47.2188167	52.9702921	39.9967568
H	47.0160230	51.9617278	39.9669487
C	46.2897190	53.9655265	39.7913275
H	45.2452156	53.7353146	39.6190305
C	45.8178901	46.9423682	32.4079861
H	46.4824974	46.7543203	31.5494267
H	44.7979179	47.0632935	32.0229750
N	46.2740783	48.1049379	33.1479025
H	47.1833100	47.9439164	33.6608055
C	45.4874624	49.0573927	33.6653243
N	44.2207133	49.2504488	33.2340167
H	43.9537859	48.9555457	32.3014777
H	43.7371187	50.0634822	33.6056387
N	45.9557647	49.8157769	34.6520749
H	46.9014079	49.6435690	35.0654184
H	45.4131709	50.6327426	34.9250195
S	49.0262232	48.3275791	44.8412258
N	47.5858900	47.3579437	42.5682435
C	47.8962691	46.0500796	42.4030014
C	46.2229972	47.3898493	42.8077965
C	46.7179939	45.2083642	42.5451815
C	45.6554520	46.0389847	42.8113455
C	45.5162410	48.5692396	42.9891420

H	44.4373712	48.5037145	43.1214583
C	46.0287134	49.8819325	43.0292005
N	47.3501030	50.1924280	43.0096259
C	47.4210239	51.5651884	43.1379984
C	46.0887194	52.1297729	43.2076552
C	45.2100583	51.0767874	43.1483615
C	48.6130219	52.2807529	43.1529891
H	48.5545779	53.3399097	43.3910729
C	49.8921991	51.7832163	42.8608115
C	51.0756216	52.5995696	42.6897702
C	51.5253605	50.4205744	42.4034411
N	50.1716050	50.4653740	42.6482072
C	52.1073328	51.7472018	42.4148358
C	52.2425862	49.2636008	42.1495688
H	53.3180870	49.3907631	42.0271446
C	51.7288097	47.9667567	41.9920812
C	52.5149267	46.8201673	41.5885032
N	50.4122593	47.6422268	42.1007024
C	50.3405604	46.2838517	41.8395653
C	49.1842186	45.5598052	42.0971246
H	49.2799735	44.4817046	42.1086412
C	51.6480276	45.7575708	41.4469194
Fe	48.8700797	48.9136132	42.6050332
O	48.4197147	49.3599742	40.8016425
H	50.3460166	48.2111188	39.9091840
O	50.0071516	48.8029136	39.2182740
O	48.4101990	49.2719303	35.7514657
C	48.9039944	48.1487953	35.4390984
O	48.4728102	47.4171706	34.5229622
C	50.1474753	47.6779712	36.2375883
C	51.2795028	48.7348484	36.2714137
C	52.5225594	48.3735223	37.1331432
C	53.5878780	49.4963967	37.2472323
H	50.2206968	50.5560280	38.9584946
O	47.5186383	51.1496440	37.4186918
H	47.9105935	50.3839462	36.9376373
H	47.0128096	50.7347985	38.1585865
O	49.9778874	51.4923729	38.7771040
H	49.1730517	49.7686474	40.3277578
H	49.1406610	51.4009519	38.2697469
H	46.0292778	57.1334215	40.3085267
H	45.8429737	46.0462326	33.0279691
H	49.5331300	49.3689096	45.5915748
H	46.7083110	44.1481667	42.2922891
H	44.6074382	45.8276001	43.0235974
H	45.8554159	53.1923301	43.2755442
H	44.1247382	51.1174236	43.0561870
H	51.1322308	53.6874567	42.7267037
H	53.1492613	51.9873686	42.2032417
H	53.5959531	46.8399021	41.4504470
H	51.9675888	44.7920768	41.0547854
H	49.8312568	47.4924489	37.2640651
H	50.4978971	46.7294084	35.8307709
H	51.5955827	48.9597257	35.2527917
H	50.8455932	49.6602174	36.6502037
H	52.1773755	48.1084697	38.1324741
H	52.9891385	47.4691309	36.7426982
H	54.4437587	49.1515170	37.8273990
H	53.9751412	49.7650379	36.2644168
H	53.2091512	50.4065001	37.7123612

Cpd I

C	46.5816208	56.5891454	39.5662195

H	45.9842330	56.6046880	38.6395921
H	47.5241272	57.1136527	39.3406259
C	46.9549822	55.1745482	39.8852290
N	48.2836353	54.8369137	40.1217901
H	49.0314542	55.5243259	40.3150038
C	48.4196449	53.5106587	40.1914615
H	49.3489504	52.9454079	40.2465340
N	47.2090094	52.9802515	40.0279036
H	47.0034258	51.9721724	40.0076966
C	46.2801619	53.9805920	39.8395233
H	45.2286022	53.7574513	39.7032468
C	45.8467761	46.9373381	32.4412460
H	46.5172141	46.7620197	31.5842168
H	44.8308803	47.0758301	32.0507937
N	46.3050477	48.0752580	33.2184263
H	47.1676145	47.8752062	33.7908658
C	45.5232081	49.0480542	33.7080682
N	44.2755579	49.2687113	33.2387635
H	44.0246395	48.9678159	32.3038178
H	43.8079221	50.1025321	33.5849292
N	45.9811153	49.7984045	34.7051635
H	46.9152525	49.6147566	35.1365345
H	45.4417865	50.6161183	34.9819379
S	48.9013770	48.3210503	44.8860198
N	47.5094008	47.3310798	42.6015195
C	47.8275878	46.0188151	42.4301666
C	46.1533832	47.3606740	42.8403526
C	46.6483222	45.1805360	42.5767938
C	45.5867326	46.0120484	42.8443911
C	45.4426306	48.5447333	43.0221399
H	44.3646760	48.4787199	43.1606516
C	45.9552475	49.8467394	43.0505345
N	47.2861986	50.1569401	43.0043555
C	47.3626150	51.5242934	43.1301996
C	46.0346806	52.0939013	43.2247461
C	45.1491506	51.0452221	43.1879050
C	48.5587479	52.2434225	43.1338179
H	48.5007681	53.3034376	43.3681787
C	49.8304151	51.7473383	42.8533375
C	51.0201720	52.5588141	42.6928440
C	51.4680239	50.3769715	42.4276717
N	50.1160556	50.4214391	42.6518049
C	52.0519863	51.7023076	42.4351585
C	52.1928001	49.2131167	42.1960514
H	53.2710119	49.3376629	42.0947154
C	51.6753589	47.9270793	42.0352734
C	52.4508079	46.7755234	41.6260409
N	50.3510998	47.6062350	42.1417428
C	50.2691898	46.2614975	41.8675635
C	49.1064158	45.5344798	42.1222288
H	49.2044095	44.4564737	42.1293501
C	51.5733341	45.7252650	41.4721367
Fe	48.7834923	48.8960301	42.4935698
O	48.4501102	49.1614240	40.8815356
H	51.1935896	49.0972920	39.9225195
O	50.4648166	49.1503214	39.2876404
O	48.4308548	49.2701736	35.8519621
C	48.8880921	48.1178459	35.5960274
O	48.3912122	47.3220082	34.7717786
C	50.1807680	47.6934777	36.3461977
C	51.3011678	48.7610113	36.2785895
C	52.6174404	48.4025570	37.0262276

C	53.6560849	49.5518032	37.1198487
H	50.1867737	50.8244143	38.8024106
O	47.3698662	51.2137574	37.3602172
H	47.8248991	50.4588427	36.9225798
H	46.9478442	50.7991277	38.1510680
O	49.9613114	51.7285774	38.4822624
H	49.6551321	49.0754978	39.8803832
H	49.0836108	51.6079140	38.0637628
H	46.0298624	57.1434168	40.3254382
H	45.8556682	46.0288359	33.0434185
H	49.4588728	49.3446794	45.6247546
H	46.6361973	44.1208549	42.3218504
H	44.5384495	45.8013033	43.0559473
H	45.8075603	53.1579837	43.2896456
H	44.0618872	51.0898996	43.1252219
H	51.0815966	53.6466046	42.7245610
H	53.0950265	51.9384441	42.2245075
H	53.5317042	46.7845080	41.4858635
H	51.8848342	44.7606058	41.0715527
H	49.9292025	47.5293715	37.3939815
H	50.5186180	46.7333968	35.9561091
H	51.5263535	48.9838573	35.2356618
H	50.8943086	49.6840240	36.6916032
H	52.3622689	48.0659261	38.0310332
H	53.0814300	47.5370895	36.5532088
H	54.5502868	49.2132671	37.6431682
H	53.9836720	49.8568432	36.1260155
H	53.2862631	50.4449840	37.6233634
